# Regional patterns of increasing Swiss needle cast impacts on Douglas‐fir growth with warming temperatures

**DOI:** 10.1002/ece3.3573

**Published:** 2017-11-18

**Authors:** E. Henry Lee, Peter A. Beedlow, Ronald S. Waschmann, David T. Tingey, Steven Cline, Michael Bollman, Charlotte Wickham, Cailie Carlile

**Affiliations:** ^1^ U.S. Environmental Protection Agency Corvallis OR USA; ^2^ Department of Statistics Oregon State University Corvallis OR USA; ^3^ Missouri Department of Natural Resources Jefferson City MO USA

**Keywords:** climate change, dendroecology, forest diseases, *Phaeocryptopus gaeumannii*, *Pseudotsuga menziesii*

## Abstract

The fungal pathogen, *Phaeocryptopus gaeumannii*, causing Swiss needle cast (SNC) occurs wherever Douglas‐fir is found but disease damage is believed to be limited in the U.S. Pacific Northwest (PNW) to the Coast Range of Oregon and Washington (Hansen et al., *Plant Disease*, 2000, *84*, 773; Rosso & Hansen, *Phytopathology*, 2003, *93*, 790; Shaw, et al., *Journal of Forestry*, 2011, *109*, 109). However, knowledge remains limited on the history and spatial distribution of SNC impacts in the PNW. We reconstructed the history of SNC impacts on mature Douglas‐fir trees based on tree‐ring width chronologies from western Oregon. Our findings show that SNC impacts on growth occur wherever Douglas‐fir is found and is not limited to the coastal fog zone. The spatiotemporal patterns of growth impact from SNC disease were synchronous across the region, displayed periodicities of 12–40 years, and strongly correlated with winter and summer temperatures and summer precipitation. The primary climatic factor limiting pathogen dynamics varied spatially by location, topography, and elevation. SNC impacts were least severe in the first half of the 20th century when climatic conditions during the warm phase of the Pacific Decadal Oscillation (1924–1945) were less conducive to pathogen development. At low‐ to mid‐elevations, SNC impacts were most severe in 1984–1986 following several decades of warmer winters and cooler, wetter summers including a high summer precipitation anomaly in 1983. At high elevations on the west slope of the Cascade Range, SNC impacts peaked several years later and were the greatest in the 1990s, a period of warmer winter temperatures. Climate change is predicted to result in warmer winters and will likely continue to increase SNC severity at higher elevations, north along the coast from northern Oregon to British Columbia, and inland where low winter temperatures currently limit growth of the pathogen. Our findings indicate that SNC may become a significant forest health problem in areas of the PNW beyond the coastal fog zone.

## 1 INTRODUCTION

Swiss needle cast (SNC) is a foliar disease caused by the ascomycete fungus *Phaeocryptopus gaeumannii* (Rhode) Petrak that is specific to Douglas‐fir (*Pseudotsuga menziesii* (Mirb.) Franco, *P. menziesii* var. *glauca* [Beissn.] Franco, and *Pseudotsuga macrocarpa (Vasey) Mayr*). *P. gaeumannii* is indigenous in western North America and occurs wherever its host is found (Boyce, 1940). In its native distribution range, epidemic outbreaks of SNC are most severe within the coastal fog zone in Oregon, Washington, and British Columbia and have steadily increased in severity since 1980 (Black, Shaw, & Stone, 2010; Hansen et al., 2000; Omdal & Ramsey‐Kroll, 2010; Shaw, Filip, Kanaskie, Maguire, & Littke, 2011). The affected area with visible SNC symptoms—chlorosis and premature needle loss—seen from annual aerial surveys of coastal Oregon has set new record highs each of the last 6 years (Ritóková et al., 2016). In the 20th century, *P. gaeumannii* has emerged in Douglas‐fir plantations in many parts of the world including much of Europe, Chile, Australia, and New Zealand (Buchwald, 1940; Kimberley, Hood, & Knowles, 2011; Lanier, 1966; Marks & Pederick, 1976; Osorio, 2007; Peace, 1962). There is mounting concern that SNC is increasing in severity, frequency, and range in association with rising winter temperatures and spring/summer precipitation and will continue to intensify over the 21st century due to climate change (Watt, Stone, Hood, & Manning, 2011; Zhao, Mainwaring, Maguire, & Kanaskie, 2011).

While the epidemiology of SNC, genetic diversity and mechanisms of pathogenicity of *P. gaeumannii* on Douglas‐fir have been well studied in young plantations, knowledge remains limited on the history and spatial distribution of SNC impacts on mature trees in the U.S. Pacific Northwest (PNW) and elsewhere. *Phaeocryptopus gaeumannii* has long believed to have been pervasive but innocuous in Douglas‐fir forests prior to 1950 (Boyce, 1940; Hood, 1982; Peace, 1962). Increased severity since ~1950 is thought to be at least in part, climate‐mediated because the causal fungus is sensitive to small differences in temperature and moisture (Black et al., 2010; Manter, Reeser, & Stone, 2005; Stone, Coop, & Manter, 2008). In coastal Oregon, a recent dendrochronological study indicates that SNC has affected periodically Douglas‐fir growth at least back to 1592, which was the earliest of the available tree‐ring records (Lee, Beedlow, Waschmann, Burdick, & Shaw, 2013). SNC impacts as measured by tree‐ring width peaked in 1984–1986—thought to be a period when the fungal population reached epidemic levels following several decades of environmental conditions favorable to growth and reproduction of *P. gaeumannii* (Lee et al., 2013).

Growth reduction in Douglas‐fir due to SNC in the PNW is symptomatic in the Coast Range of Oregon and Washington (Hansen et al., 2000; Rosso & Hansen, 2003). In the most heavily affected 10‐ to 30‐year‐old plantations in north coastal Oregon, height and basal area growth were reduced by ~25% and 35%, respectively, during a SNC outbreak in the mid‐1990s (Maguire, Kanaskie, Voelker, Johnson, & Johnson, 2002). In a highly affected mature forest stand near Tillamook, Oregon, SNC reduced growth of 80‐year‐old Douglas‐fir trees by 85% during severe SNC outbreaks with some trees showing 10 or more years having no observable growth (Black et al., 2010). At less diseased sites in coastal Oregon, annual radial stem growth of mature Douglas‐fir was reduced by 18%–28% on average during SNC outbreaks between 1590 and 2011 (Lee et al., 2013).

While SNC symptoms are often noted in plantations in Northern Idaho and Western Montana (Hagle, Gibson, & Tunnock, 2003), there have been few broad‐scale studies to quantify the impact of SNC on tree growth outside of the coastal fog zone in the PNW. A comprehensive study involving 59 young Douglas‐fir stands (10–23 years) found no growth reductions in the Oregon Cascades during a SNC outbreak between 2001 and 2006 (Filip et al., 2007). This study assessed the SNC impact by observing natural outbreaks but this is difficult to do in mature forest stands where the history of SNC outbreaks is unknown and must be inferred. A previous dendrochronological study (Lee et al., 2016), using earlywood (EW) and latewood (LW) ring width chronologies, showed that the ubiquitous SNC affected Douglas‐fir growth across a longitudinal transect from the west side of the Coast Range to the west slopes of the Cascade Range of Oregon. Air temperature and dewpoint deficit (DPD) were the most important climate factors affecting Douglas‐fir growth, and soil moisture and SNC modified the growth response to these climate factors.

Lee et al. (2016) examined the growth‐climate relations for mature Douglas‐fir by adjusting the tree‐ring width series for SNC but did not reconstruct the local and regional history of SNC impacts on radial stem growth. We extend the findings of Lee et al. (2013, 2016) to determine the impact of SNC across a diversity of ecoregions in the PNW ranging from wet maritime in the Coast Range to dry Mediterranean in the Cascade Range. The key growth pattern in tree‐ring records associated with SNC of coastal Douglas‐fir is a sinusoidal cycle of anomalously low growth having a primary periodicity of ~20–30 years and a harmonic periodicity of ~4 years (Lee et al., 2013). The cyclical patterns of SNC impact on Douglas‐fir growth occur throughout the life of the tree and because of the effects of synoptic seasonal weather patterns on fungal growth, are synchronous across coastal Oregon.

Three major phases of the infection cycle of *P. gaeumannii* are relevant to the understanding of the climate‐disease relation and history of SNC outbreak events (Manter et al., 2005): (1) *P. gaeumannii* reproduces by ascospores and pseudothecia (i.e., fruiting bodies) proliferate in winter from December to April; (2) sporulation occurs in synchrony with bud break and shoot elongation from May to July and only current‐year needles are infected (Hood & Kershaw, 1975; Stone, Capitano, & Kerrigan, 2008); and (3) needle colonization by hyphal growth on the needle surface into the stomata occurs year round following initial infection. Because released spores can dessicate and lose viability within several days, leaf wetness at time of sporulation is important for ascospore germination (Manter et al., 2005; Stone, Coop, et al., 2008; Watt et al., 2011). Within the SNC impact zone along the coast of Oregon and Washington, disease severity is associated with a combination of mild winter temperatures and high leaf wetness in spring (Manter et al., 2005; Stone, Coop, et al., 2008) as well as mild summer temperatures ranging between the optimum temperatures for ascospore germination and germ tube growth at 18°C and 22°C, respectively (Capitano, 1999). These climatic conditions for disease severity have also been verified in New Zealand (Watt et al., 2011). The maturation period for *P. gaeumannii* ranges from 1 to 2 years in some areas of the Coast Range to 4–7 years in the Cascade Range of Oregon and Washington as evidenced by pseudothecia on young and older needles, respectively (Stone, Capitano, et al., 2008). The low‐ to higher‐frequency variations in the tree‐ring width series of coastal Douglas‐fir have been attributed to the slow buildup of fungal abundance over multiple generations of *P. gaeumannii* (Lee et al., 2013).

This study reconstructs the regional history of SNC impacts on Douglas‐fir growth for nine sites in western Oregon differing in site conditions, elevation, topography, and proximity to the coast based on master chronologies of EW and LW ring widths of the host and nonhost tree species. Specific objectives of this research were to test the following hypotheses: (1) SNC is sensitive to winter and summer temperature, and summer precipitation, and so, spatial variability in SNC severity can be attributed to variations in site conditions, location, and elevation; (2) SNC impacts display primary periodicities of 20–30 years and secondary periodicities of 4–6 years as seen for the coast sites (Lee et al., 2013); and (3) winter temperature is a more limiting factor of *P. gaeumannii* than summer conditions in the high Cascades and vice versa for low‐ to midelevation sites. We hypothesized that the impacts of SNC should be synchronous at low‐ to mid‐elevations where summer conditions are more limiting to fungal dynamics than winter conditions but delayed at higher elevations in the Cascade Range where winter conditions are less favorable to *P. gaeumannii*.

## 2 METHODS

### 2.1 Research sites

**Figure 1:**
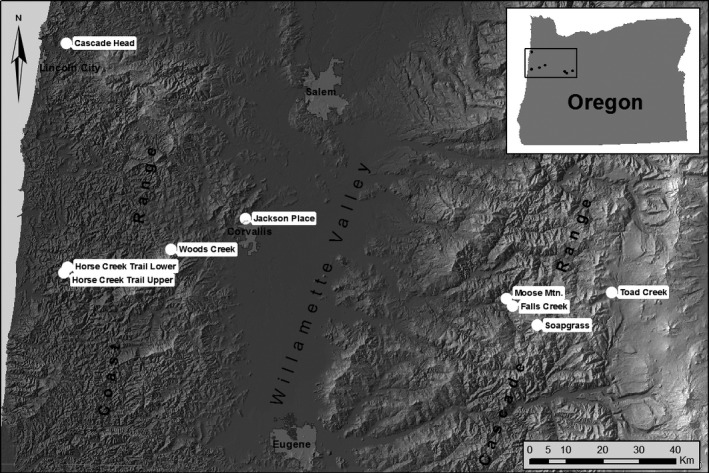
Tree core samples were collected from nine field sites located in mature Douglas‐fir stands on the west and east sides of the Coast Range, in the Willamette Valley, and on the west side of the Cascade Range (Lee et al., 2016)

Nine mature, closed‐canopy forest stands dominated by Douglas‐fir were located on state and federal lands in western Oregon, USA (Figure 1). The nine stands represent a range of climatic and edaphic conditions at varying elevations and proximal distances to the coast (Table 1). Douglas‐fir for all study sites displayed SNC symptoms of sparse canopies, loss of older needles, and chlorotic needles infected by *P. gaeumannii* in 2014. Dwarf mistletoe (*Arceuthobium* spp.) was widespread in western hemlock (*Tsuga heterophylla*) at Horse Creek Trail Lower and Soapgrass Mountain but was absent at drier sites. Douglas‐fir dwarf mistletoe (*Arceuthobium douglasii*) was not observed at any site. As western hemlock is not a host of *P. gaeumannii*, SNC affects the growth of the two tree species differently. The sites will be referred to by their abbreviations as follows: CH = Cascade Head; HCTL & HCTU = Horse Creek Trail Lower & Upper; WC = Woods Creek; JP = Jackson Place; FC = Falls Creek; MM = Moose Mountain; SG = Soapgrass; TC = Toad Creek.

**Table 1:** Site information for tree core sampling in western Oregon (Lee et al., 2016)

Name	Latitude, longitude	Elevation (m)	Distance to coast (km)	Aspect	Slope (%)	Basal area density (m^2^/ha)	Mean daily minimum plant available soil moisture (mm) and percent of maximum in parentheses^a^	Climate and edaphic grading
Cascade Head (CH)	N45°02′ W123°54′	150	8.7	80° (E)	19	88^b^	93.9 (39%)	Temperate rainforest
Horse Creek Trail Lower (HCTL)	N44°29′ W123°54′	280	13.4	320° (NW)	29	69^c^	NA	Temperate rainforest
Horse Creek Trail Upper (HCTU)	N44°28.5′ W123°55′	470	14.8	310° (NW)	26	98^c^	NA	Temperate rainforest
Woods Creek (WC)	N44°32′ W123°33′	525	42.0	330° (NW)	6	72^c^	NA	Warm, dry valley
Jackson Place (JP)	N44°36.7′ W123°17.5′	180	61.2	30° (NE)	28	67^c^	NA	Warmer, drier valley
Falls Creek (FC)	N44°24′ W122°22′	530	136.6	45° (NE)	8	60^b^	33.7 (23%)	Warm, dry montane
Moose Mountain (MM)	N44°25′ W122°24′	658	134.8	250° (SW)	17	61^b^	13.2 (17%)	Warm, drier montane
Soapgrass Mountain (SG)	N44°21′ W122°17′	1,190	144.0	250° (SW)	6	148^b^	36.0 (21%)	Cool, wet montane
Toad Creek (TC)	N44°26′ W122°02′	1,198	163.1	100° (E)	12	85^b^	26.8 (32%)	Cool, dry montane

a Mean values for the years 1998–2009 from Beedlow et al. (2013).

b Basal area density is based on a complete plot survey of individual trees at these permanent field sites.

c Basal area density is based on multiple stand measurements using a wedge prism that has a basal area factor of either 20 or 30.

The coast sites (CH, HCTL, HCTU) are situated within the coastal fog zone where humidity and precipitation levels are highest with total annual precipitation for CH averaging 2,041 mm/year, whereas the valley sites (WC, JP) are situated in the rainshadow of the Coast Range and represent the driest sites with annual precipitation totals for JP averaging 1,236 mm/year. As Pacific air masses move eastwards to the Cascade Range, humidity and precipitation levels increase slightly, with annual precipitation totals for FC and SG averaging 1,708 and 2,014 mm/year, respectively. This region experiences a Mediterranean climate regime characterized by an annual summer drought and moist‐cool winters with 89% of total annual precipitation falling between October and May. See Beedlow, Lee, Tingey, Waschmann, and Burdick (2013) and Lee, Tingey, Beedlow, Johnson, and Burdick (2007), Lee et al. (2013, 2016) for a complete description of the sites.

### 2.2 Dendrochronological and climate data

Tree core samples from 17 to 29 dominant and codominant Douglas‐fir trees and 4 to 16 western hemlock trees were taken at approximately breast height (1.4 m) using 5‐mm diameter increment borers, collecting one or two cores per tree at each site (Lee et al., 2016) (Table 2). The cores were air‐dried, mounted, sanded, and digitized using a color flatbed scanner. EW and LW tree‐ring widths were measured to the nearest 0.01 mm using WinDENDRO 2008g software (Regent Instruments Inc., Quebec, Canada). The individual ring width time series were visually cross‐dated and verified using the program COFECHA (Holmes, 1983) to ensure the correct calendar year was assigned to each ring. The interseries correlation ranged from 0.46 to 0.55 for Douglas‐fir and 0.12 to 0.39 for western hemlock (Table 2). The mean sensitivity ranged from 0.16 to 0.26 for Douglas‐fir and 0.18 to 0.32 for western hemlock. The EW and LW time series were log transformed and detrended using either a cubic spline smoother with a 50% frequency response of 32 years or a horizontal line to remove the age‐related trend in the initial ~70 years. Master chronologies of EW and LW ring widths for the nine sites were calculated as the median of the standardized time series for individual trees.

The Douglas‐fir master chronologies by Lee et al. (2016) were used to infer the seasonal effects of temperature, water, and disturbances on intra‐annual tree growth using climate data from several sources. Divergences between the master chronologies of Douglas‐fir and western hemlock were used as auxiliary information to detect outliers in the Douglas‐fir chronologies associated with forest disturbance agents. The divergence between the observed and climate‐based model prediction of Douglas‐fir growth is the primary data source to infer the history of forest disturbances because the western hemlock chronologies are not pure climate proxies free of disturbance. In this study, we reconstruct the history of forest disturbances in Douglas‐fir using growth‐climate relations similar but more parsimonious to those reported by Lee et al. (2016) and the western hemlock chronologies.

**Table 2:** Site and tree core sampling information and dendrochronology summary statistics for Douglas‐fir (Lee et al., 2016) and western hemlock

Name	Species	No. of trees cored	Pith date^a^	Interseries correlation^b^	Mean sensitivity^c^
Cascade Head (CH)	Douglas‐fir	17	1870	0.46	0.26
	Western hemlock	6	1900	0.19	0.32
Horse Creek Trail Lower (HCTL)	Douglas‐fir	9	1586^d^	0.50	0.26
	Douglas‐fir	8	1860^d^		
	Western hemlock	15	1905	0.12	0.26
Horse Creek Trail Upper (HCTU)	Douglas‐fir	16	1859	0.47	0.20
	Western hemlock	5	1883	0.39	0.18
Woods Creek (WC)	Douglas‐fir	20	1873	0.52	0.16
	Western hemlock	16	1873	0.31	0.23
Jackson Place (JP)	Douglas‐fir	19	1874	0.54	0.19
Falls Creek (FC)	Douglas fir	26	1890	0.53	0.16
	Western hemlock	4	1929	0.13	0.22
Moose Mountain (MM)	Douglas‐fir	24	1903	0.51	0.17
Soapgrass Mountain (SG)	Douglas‐fir	29	1547	0.51	0.18
	Western hemlock	14	1864	0.24	0.22
Toad Creek (TC)	Douglas‐fir	18	1811	0.55	0.16
	Western hemlock	8	1864	0.44	0.20

a Oldest year.

b The average correlation between each detrended time series and the mean of all other detrended time series.

c The mean absolute first difference of each detrended time series relative to its running mean value. The index ranges from 0 (i.e., no variability) to 2 (i.e., high variability with periodicity of 2 years).

d The forest stand contained two different‐aged cohorts of Douglas‐fir growing in adjacent sections.

Monthly mean maximum, minimum, and dewpoint temperatures and total precipitation data for the period 1895–2012 were obtained from the PRISM Climate Group at Oregon State University at http://prism.oregonstate.edu. The divisional monthly Palmer Drought Severity Index (PDSI) for the period 1895–2012 was obtained from the National Oceanic and Atmospheric Administration's National Climate Data Center at http://www.ncdc.noaa.gov. Variables were obtained for the specific study site locations when spatially interpolated climate data were available and for the nearest location or division otherwise (Lee et al., 2016). DPD was calculated from monthly PRISM data as the difference between mean air temperature and the dewpoint temperature and is a measure of evaporative demand. The climate variables used in the time series model were daily maximum air temperature, total precipitation, DPD, and PDSI which were summarized as seasonal averages that corresponded best with intra‐annual growth.

### 2.3 Time‐series intervention analysis

Following Cook (1985, 1987), the master chronology (Y_*t*_) has been described by Lee et al. (2016) as having a mean response function with components for climate (C_*t*_) and disturbance (D_*t*_), that is, *E*(Y_*t*_) = C_*t*_ + D_*t*_. The climate component, C_*t*_, represents the interactions of temperature, precipitation, soil moisture, and evapotranspiration demand on tree growth and is assumed to be the same for all trees within a stand (Fritts, 1976). The disturbance component, D_*t*_, has been described by Lee et al. (2016) as a set of pulse functions to detect outliers representing the outbreak events of one or more forest disturbance agents. Maximum likelihood for a Gaussian time series was used for parameter estimation and hypothesis testing to reconstruct the forest disturbance history of SNC outbreaks by site for the years 1895–2011. Given the explicit characterization of the growth‐climate relation (C_*t*_) of the host species (Lee et al., 2016), the disturbance events are unknown and must be inferred by comparing Y_*t*_ and C_*t*_ to detect growth divergences which cannot be attributed to the measured climate variables. Outlier detection tests are performed using the Wald test statistic, that is, $W={\hat{\beta }}_{i}/{}^{2}\sqrt{\hat{\text{Var}}\left({\hat{\beta }}_{i}\right)}$ where β_*i*_ is the model parameter for the pulse disturbance event at time *t* = *t*_*i*_ for *i* = 1, 2, … r. In general, the disturbance events t* = (*t*_1_, *t*_2_, …, *t*_*r*_) are unknown and must be inferred. Some multiple testing adjustments such as Bonferroni or Tukey and/or specification of a critical value *A* are appropriate for detection of multiple outliers because every point is a potential outlier. Chang and Tiao (1983) suggest values of *A* between 3 and 4. The forest disturbance events identified by Lee et al. (2016) were improved upon through iteration and perturbation based on the Wald test statistic using a greater critical value *A* of 4 for the reconstruction of SNC impacts by site. See Lee, Wickham, Beedlow, Waschmann, and Tingey (2017) for a more complete description of the time‐series intervention methodology.

### 2.4 Swiss needle cast index of impact on stem growth

To reconstruct the site‐specific history of SNC impact, divergences between the observed and climate‐based model predictions were detected as outliers based on statistical fit of the TSIA model with pulse interventions. Growth anomalies that could not be attributed to climate were modeled by a pulse intervention event. For the diseased years specific to Douglas‐fir, the SNC index of impact was equal to one minus the negative pulse intervention parameter backtransformed to the original scale (Lee et al., 2016). For example, a parameter value of −1 corresponds to a SNC index value of 1–10^−1^ = 0.9 or a 90% reduction in growth. The SNC index of impact was defined to be 0 for the other years.

### 2.5 Canonical correlation analysis

Once the history of forest disturbance was reconstructed for each site, we cross‐correlated the SNC index with seasonal averages of temperature and precipitation (Lee et al., 2013). The PRISM monthly climate data were summarized as seasonal averages of temperature and precipitation that corresponded to the three major phases of the infection cycle (Manter et al., 2005). Winter temperature was calculated as average daily maximum temperature for one or more months between December and March. Spring/summer precipitation and temperature were calculated as total precipitation and mean daily maximum temperature for one or more months between May and July. Cross‐correlation and canonical correlation analyses were used to identify the weighted moving averages of current and past seasonal temperature and precipitation values that optimize the correlation with SNC impact.

### 2.6 Spectral analysis

For sites in coastal Oregon, the tree‐ring width chronologies for mature Douglas‐fir were synchronous and displayed cyclical patterns having primary periodicities of 20–30 years and secondary periodicities of 4–6 years that could not be attributed to climate (Lee et al., 2013). These concurrent sinusoidal patterns in Douglas‐fir stem growth adjusted for climate were attributed to SNC within the coastal fog zone (Lee et al., 2013). This recent finding has led us to expand our scope to detect SNC impacts on Douglas‐fir growth outside of the coastal fog zone. The episodic patterns of SNC impact are best examined using spectral or frequency domain analysis. Spectral analysis partitions the variance of a time series into a discrete and finite set of components, each of which is associated with a particular frequency band. The representation of a time series in the frequency domain is called the spectrum. The spectrum measures the strength of different frequency components, that is, peaks in the spectrum occur at the key frequencies. The primary frequency of a time series corresponds to the sinusoidal term that is most strongly associated with the time series, the secondary frequency with the next most strongly associated.

Cospectral analysis examines the synchronicity of SNC chronologies based on whether the spectrum at multiple sites has the same key frequencies (i.e., variations in each time series are associated with sinusoids having the same frequencies). Cospectral analysis was used to determine whether the aclimatic growth anomalies of inland Douglas‐fir displayed a cyclical pattern having a primary periodicity of 20–30 years and a secondary periodicity of 4–6 years in synchrony with the SNC index for coastal Douglas‐fir.

## 3 RESULTS

**Figure 2:**
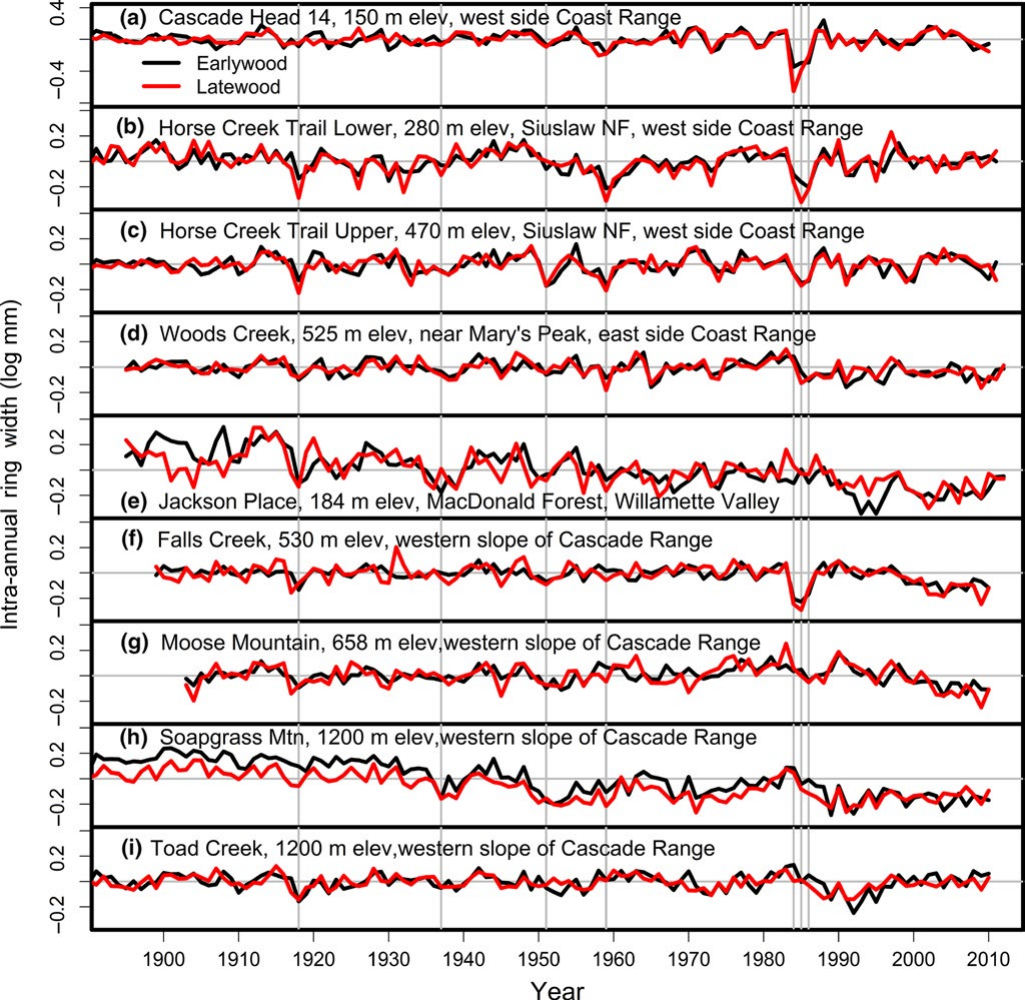
Chronologies of earlywood and latewood widths of mature Douglas‐fir across a network of sites from the Coast Range to the west side of the Cascade Range of Oregon (Lee et al., 2016). The vertical gray lines denote climatic pointer years of 1918, 1951, 1959, 1984, 1985, and 1986

All sampled stands experienced anomalously low radial stem growth that could not be accounted for by current and previous year seasonal climatic metrics used in this study. Regional synchronous patterns of growth suppression across the sites, attributable to forest disturbances, appeared in the master chronologies in 1918, 1951, 1959, and, most notably in 1984–1986 (Figure 2). For CH, HCTL, and FC, record low growth occurred in 1984 when summer PDSI was near a record high (i.e., abnormally wet summer), indicating a divergence between growth and climate. The SNC impact on tree growth was modeled as pulse interventions for the years having observed growth values that diverged significantly (*p*‐value < .05/*r* where *r* = number of outliers detected) from the climate‐based model prediction and were specific to Douglas fir. The site‐specific TSIA models with components for climate and disturbance explained between 60% and 85% of the total variation in the EW and LW width chronologies (Table A1). The disturbance component associated with SNC explained the majority of the total variation in radial stem growth for all low‐ to midelevation sites based on Kruskal's measure of relative importance (table 2 in Lee et al., 2016).

### 3.1 Pseudoperiodicities in tree growth

**Figure 3:**
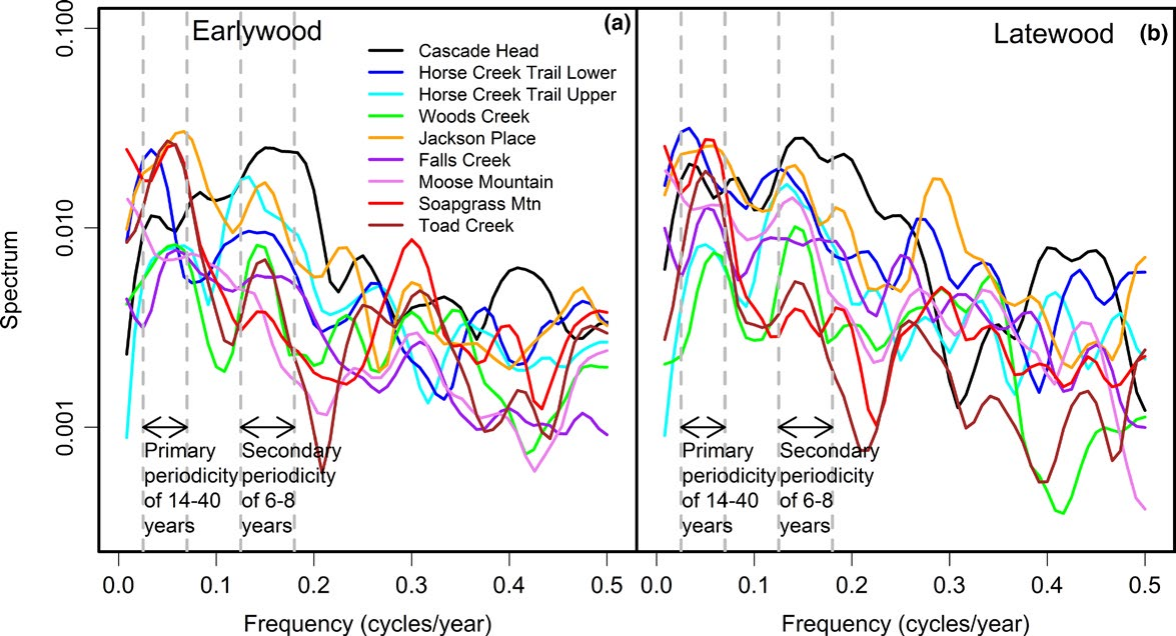
A comparison of the spectrum of (a) earlywood and (b) latewood ring width chronologies for the years 1895–2011 indicates a wide range of tree variability between growth periods within a site and between sites. The master chronologies for two coast sites, Cascade Head and Horse Creek Trail Lower, have a primary periodicity of ~40 years and a secondary periodicity of 6.2–8 years. The master chronologies for two high elevation sites, Soapgrass Mountain and Toad Creek, have a primary periodicity of ~20 years which also is indicative of a Swiss needle cast disease cycle

The time series chronologies for EW and LW ring widths displayed a mixture of low, medium, and high frequencies indicative of variability in growth response to both climate and SNC (Figure 3). One coast site, HCTL, and three Cascade sites, FC, SG, and TC, displayed strong sinusoidal patterns having a primary periodicity of 14–40 years and a secondary periodicity of 6–8 years as indicated in the frequency domain by peaks in its spectrum at a frequency of 0.25–0.07 cycles/year and 0.12–0.17 cycles/year, respectively. One low elevation coast site, CH, within the SNC impact zone displayed sinusoidal impacts on growth every 5–7 years. The pseudoperiodic patterns of growth were evident in both EW and LW width. For two inland sites, JP and MM, where evaporative demand was high during the annual summer drought, the spectrum had a primary peak at 0 frequency which was seen in the time domain as a decreasing trend in growth rates in the most recent decades when temperatures were increasing. The 470‐year‐old Douglas‐fir trees at SG displayed a slower declining growth trend since ~1905. The broad peaks in the spectrum indicated a mixture of climate and SNC effects that interacted and were confounded.

### 3.2 Outlier detection using time‐series intervention analysis

**Figure 4:**
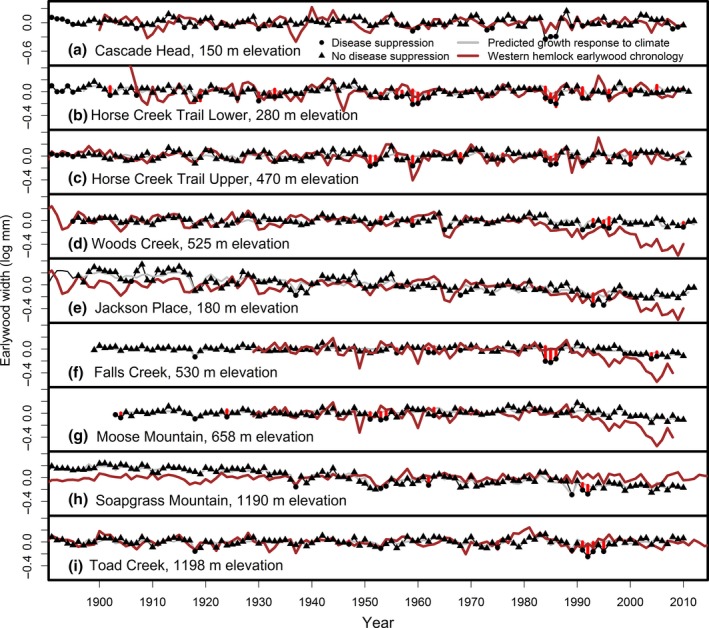
Observed and predicted earlywood width chronologies of Douglas‐fir in comparison with observed earlywood chronology of Western hemlock for the nine study sites based on time‐series intervention model. Vertical red bars represent anomalously low growth associated with Swiss needle cast (SNC) and not accounted for by the effects of climatic factors. These anomalous growth years are identified as statistically significant divergences between the host chronology (black line) and the predicted growth response to seasonal climatic factors (gray line) at the 0.05 overall level of significance. Based on the interaction of previous‐year dewpoint deficit and SNC, each year was classified into one of two disease states: (1) growth suppression due to SNC (●) and (2) no growth suppression (▲). The master chronology for Cascade Head was plotted on a different scale because the interannual variability was greater at this coast site

To reconstruct the site‐specific history of forest disturbance, the growth anomalies that could not be attributed to climate were detected as outliers based on time series analysis with pulse interventions for the disturbance years (Lee et al., 2013, 2016, 2017). An outlier is detected when the pulse intervention parameter, which represents the divergence between observed growth and the climate‐based model prediction, is statistically less than zero based on a one‐sided Wald test at an overall 0.05 level of significance with a Bonferroni correction. In the period 1895–2011, the number of disturbance years ranged from 4 (3%) to 20 (17%) for EW and 3 (3%) to 12 (10%) for LW (Table A1). Many of these growth anomalies represented a divergence between the Douglas‐fir and western hemlock chronologies, most notably in 1984–1986 (Figure 4). Several low growth anomalies were detected as outliers in both the Douglas‐fir and the western hemlock chronologies, most notably in 1917, 1918, 1937, and 1991 and consequently were not attributed to a species‐specific forest disturbance such as SNC (Table A1). Two low elevation coast sites, CH and HCTL, within the SNC impact zone experienced the most disturbance outbreak events, while a hot, dry valley site, JP, and a high Cascade site, SG, outside of the coastal fog zone experienced the fewest number of forest disturbances.

**Figure 5:**
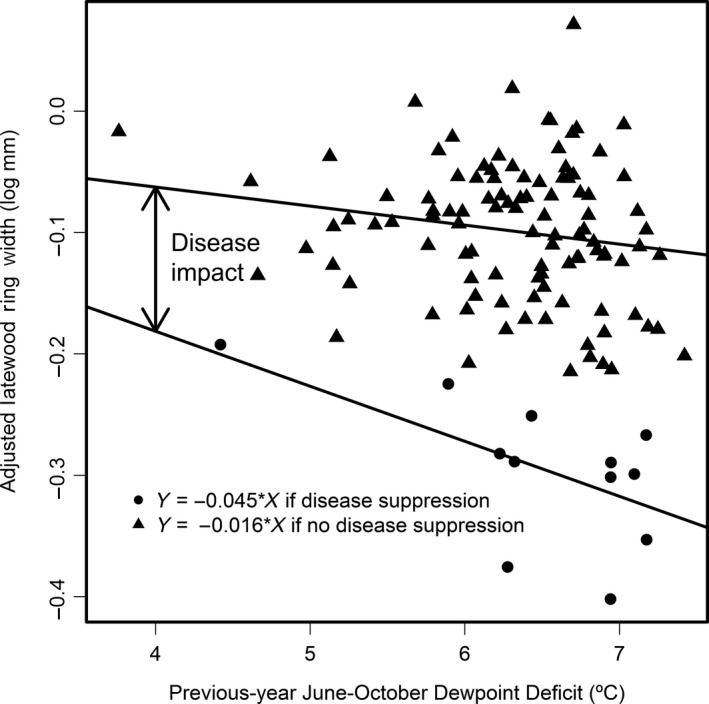
Growth response to previous‐year dewpoint deficit (pDPD) at Horse Creek Trail Lower varies depending upon the severity of Swiss needle cast disease (SNC). Growth is more sensitive to pDPD in years with high disease severity and less sensitive in years with low disease severity. The change in slope is a reliable indicator of SNC disease impact

**Figure 6:**
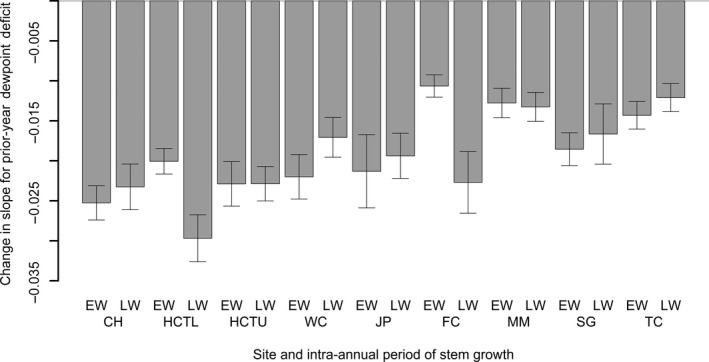
Interaction of previous‐year dewpoint deficit (DPD in °C) and Swiss needle cast (SNC) on earlywood (EW) and latewood (LW) width. Prior‐year DPD corresponds to the months of June–October (July–October at Woods Creek and Soapgrass). Growth response to prior‐year DPD varied depending upon whether growth was or was not suppressed by SNC. All changes in slope are significantly different from zero at the 0.05 level of significance. CH, Cascade Head; HCTL & HCTU, Horse Creek Trail Lower & Upper; WC, Woods Creek; JP, Jackson Place; FC, Falls Creek; MM, Moose Mountain; SG, Soapgrass; TC, Toad Creek

Examination of the negative outlier years indicated that high DPD in the previous‐year (pDPD) reduced growth more in a disturbance year than in years when disturbance was absent (Figure 5). This interaction of pDPD and disturbance was alternatively modeled as a change in slope of pDPD and resulted in a more parsimonious model having fewer pulse intervention terms and a better statistical fit based on a lower Akaike information criterion. The slope of pDPD for the disturbance outbreak years was most negative for the coastal sites where Douglas‐fir forests were more heavily infected by SNC and least negative for montane sites on the west slope of the Cascade Range of Oregon where SNC was less severe (Figure 6).

### 3.3 Reconstruction of SNC outbreaks for the period 1895–2011

**Figure 7:**
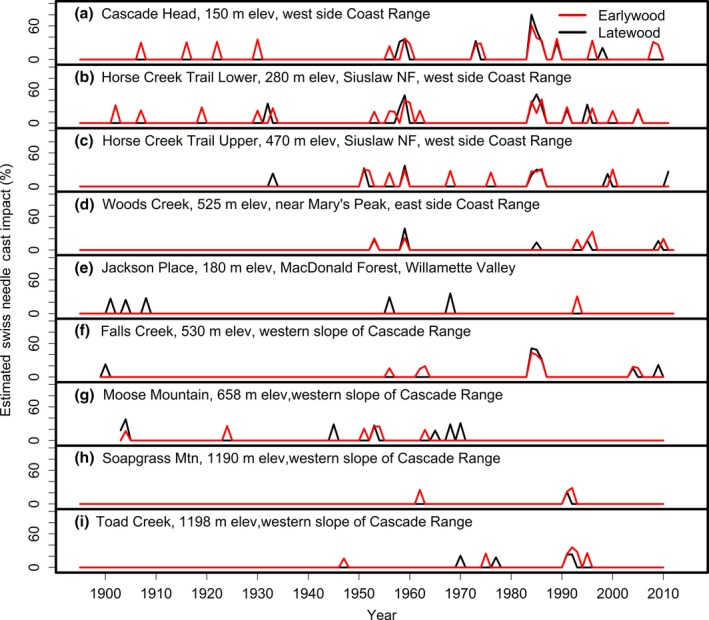
A comparison of the percent reduction in earlywood and latewood ring width increment of Douglas‐fir attributed to Swiss needle cast for nine study sites from the west slopes of the Coast Range to the west slopes of the Cascade Range of Oregon. The growth anomalies could not be explained by seasonal climate variables for temperature and water. Peak SNC impacts occurred in 1959 and 1984–1986 and were synchronous across the region

The outbreak reconstructions based on the SNC index indicated that reductions in Douglas‐fir stem growth attributable to SNC have occurred periodically in western Oregon for the period of study from 1895 to 2011 (Figure 7). Synchronous outbreaks in 1959 and 1984–1986 were inferred for four sites. Two coast sites, HCTL and HCTU, recorded synchronous outbreaks since 1895, whereas CH outbreaks were coherent after 1950. The 1984–1986 outbreak was exceptionally intense resulting in estimated reductions in EW growth of 34%–61% for CH and 31%–44% for FC. These episodic growth anomalies that could not be attributable to climate have varied in frequency and intensity but were highly synchronous and averaged 1–2 years in duration across the region from the coast to the mid‐Cascades (Table 3). The average return interval—which is the number of years between the start of two consecutive outbreaks—ranged from 7.9 to 15.0 years within the SNC impact zone and 14.6 to 48 years outside the SNC impact zone based on EW growth series (Table 3). Growth anomalies in the first half of the 20th century were not as intense nor frequent as those after 1950 (Figure 7). The number of disturbance events in EW growth ranged from 0 to 5 years for an average of 1.3 years prior to 1950 and from 1 to 13 years for an average of 6.9 years after 1950. From 1920 to 1945, site‐specific outbreaks occurred at relatively low levels, most notably for the inland sites in the Willamette Valley and Cascade Range. For the high Cascade and valley sites, the 1990s were characterized by a period of more intensive outbreaks which were not synchronous with the mid‐Cascade sites.

**Table 3:** Growth anomalies associated with a forest disturbance specific to Douglas‐fir were detected using time‐series intervention analysis and represented divergences between the observed and climate‐based model predictions that were statistically significant at the 0.05 level. The model parameters for the pulse interventions were used to infer the magnitude of the growth response to forest disturbance that could not be attributed to climate and diverged from the western hemlock chronologies. The frequency, duration, and magnitude of the tree‐ring‐based reconstructions of forest disturbance history are reported by growth period for each site

Site	Start of record	No. of outbreaks EW^a^, LW^b^	Duration (year) EW^a^, LW^b^		Return interval^c^ (year) EW^a^, LW^b^		Reduction in growth (%) EW^a^, LW^b^	
			Mean	*SD*	Mean	*SD*	Mean	*SD*
Cascade Head	1895	11, 5	1.5, 1.6	0.7, 0.9	10.1, 10.0	6.2, 24.0	34, 40	8,19
Horse Creek Trail Lower	1895	14, 6	1.3, 1,5	0.6, 0.8	7.9, 18.3	6.3, 13.2	27, 34	8,10
Horse Creek Trail Upper	1895	7, 6	1.4, 1.3	0.8, 0.8	15.0, 19.3	18.5, 10.8	28, 28	2, 6
Woods Creek	1895	5, 5	1.2, 1.0	0.4, 0.0	23.0, 22.8	23.1, 21.1	22, 21	6, 10
Jackson Place	1895	1, 5	1.0, 1.0	NA, 0.0	NA, 21.2	NA, 19.0	31, 29	NA, 4
Falls Creek	1899	4, 4	2.0, 1.5	0.8, 1.0	26.3, 27.5	21.7, 38.5	25, 32	12,15
Moose Mountain	1903	5, 6	1.2, 1.2	0.4, 0.4	14.6, 9.0	11.0, 16.5	22, 27	4, 7
Soapgrass Mountain	1895	2, 1	1.5, 1.0	0.7, NA	48.0, 96.0	26.9, NA	25, 21	3, NA
Toad Creek	1895	4,3	1.5, 1.3	1.0, 0.6	25.0, 32.0	20.5, 37.4	26, 21	6, 2

a Earlywood.

b Latewood.

c Return interval is the number of years between the start of two consecutive outbreaks.

### 3.4 Cyclical patterns of SNC

**Figure 8:**
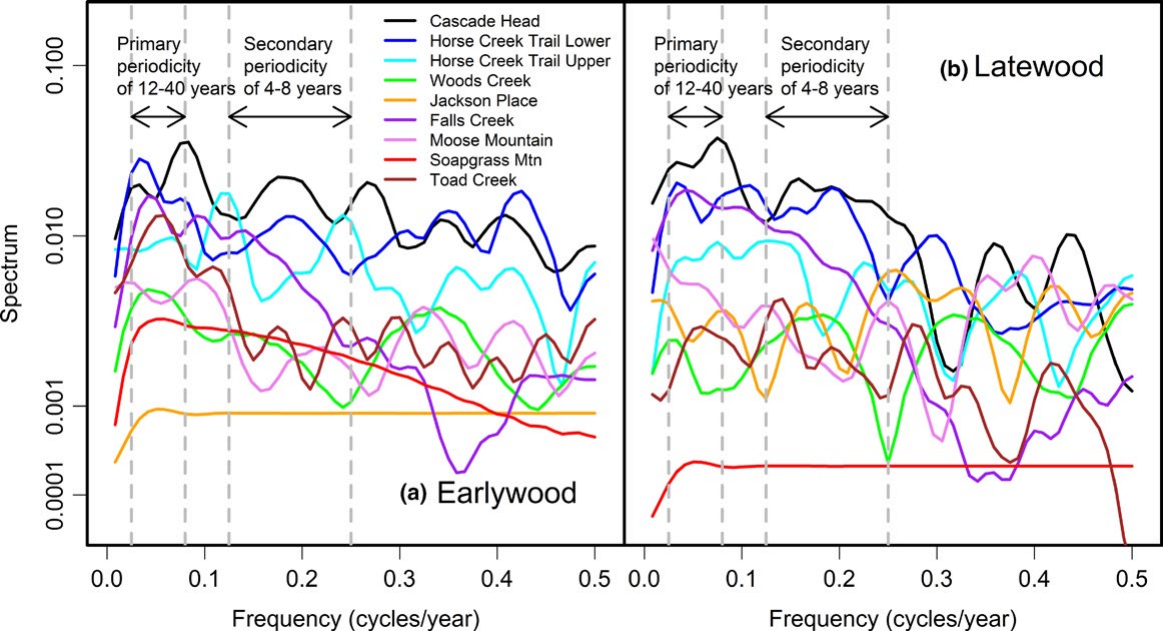
A comparison of the spectrum of the Swiss needle cast index (SNC) of impact on (a) earlywood and (b) latewood ring width for the years 1895–2011 indicates a wide range of disease severity between sites. The SNC index for two coast sites, Cascade Head and Horse Creek Trail Lower, and a high Cascade site, Toad Creek, display a primary periodicity of 12–40 years and a secondary periodicity of 4–8 years

Spectral analysis found both high‐ and low‐frequency variabilities in the climate‐adjusted SNC index for the common period 1895–2010 for six of the nine study sites (Figure 8). The spectra of the site‐specific SNC indices displayed primary peaks in the frequency range between 0.025 and 0.083 (12–40 years periodicity) and secondary peaks in the frequency range between 0.125 and 0.250 (4–8 years periodicity). The magnitude of the spectrum of the SNC index at the primary and secondary frequencies characterized the disease severity and was greatest for two coastal sites, CH and HCTL, within the SNC impact zone and least for three inland sites, JP, MM, and SG. For CH, the primary and secondary periodicities in SNC outbreaks were more frequent and regular after 1950 (data not shown) as no forest disturbance outbreaks were detected in the LW series prior to 1950 (Figure 7a). The sinusoidal nature of SNC effects on Douglas‐fir growth was more pronounced in EW than LW for all sites except JP and MM as indicated by a greater primary peak in the EW spectrum distributed across a narrower range of frequencies (Figure 8). The cyclical pattern of SNC outbreaks was more sporadic for the less severely impacted sites (JP, MM, and SG) as indicated by the absence of either a primary periodicity of 12–40 years or a secondary periodicity of 4–8 years in the spectrum of the SNC index.

**Figure A1:**
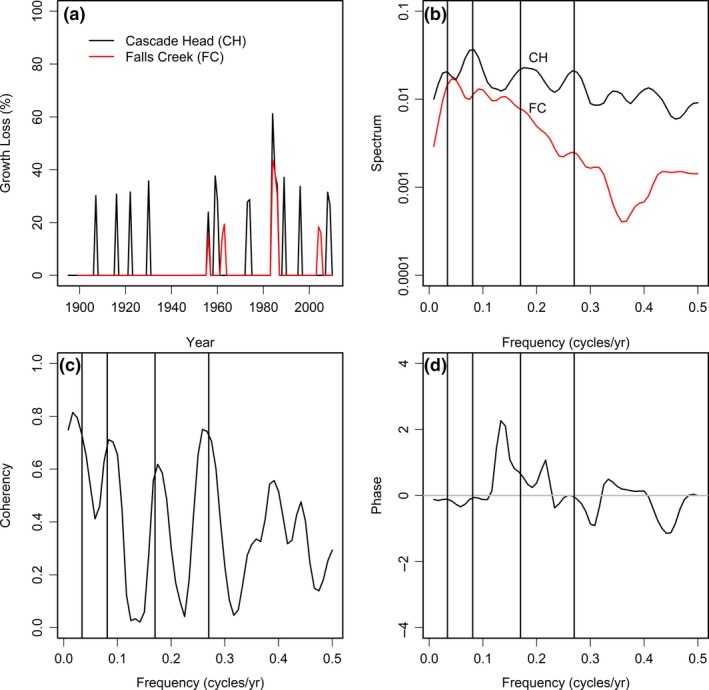
(a) Swiss needle cast indices of impact on earlywood growth for Cascade Head and Falls Creek are (c) coherent and (d) in phase at both low and high frequencies. (B) The power spectrum has a primary peak at a frequency of ~0.083 cycles/year for Cascade Head and ~0.042 cycles/year for Falls Creek

**Figure A2:**
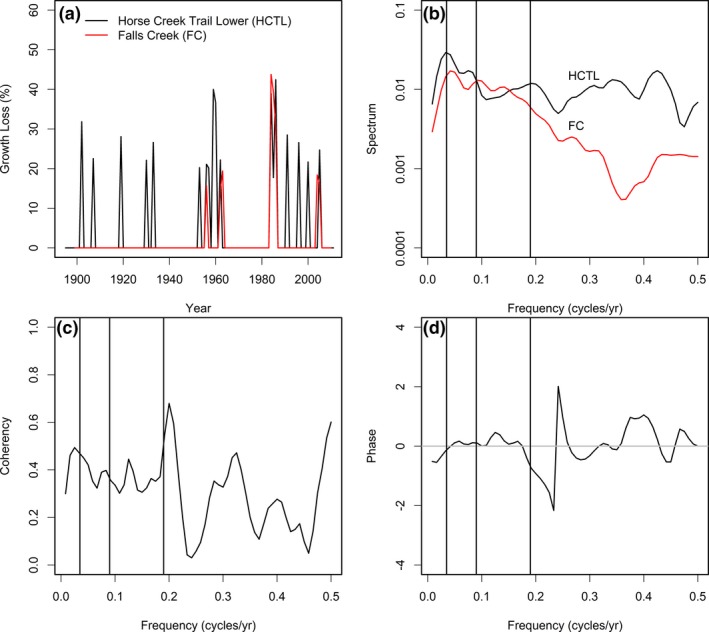
(a) Swiss needle cast indices of impact on earlywood growth for Horse Creek Trail Lower and Falls Creek are (c) coherent at both low and high frequencies and (d) in phase at high frequencies. (b) The power spectrum has a primary peak at a frequency of ~0.033 cycles/year for Horse Creek Trail Lower and ~0.042 cycles/year for Falls Creek

**Figure A3:**
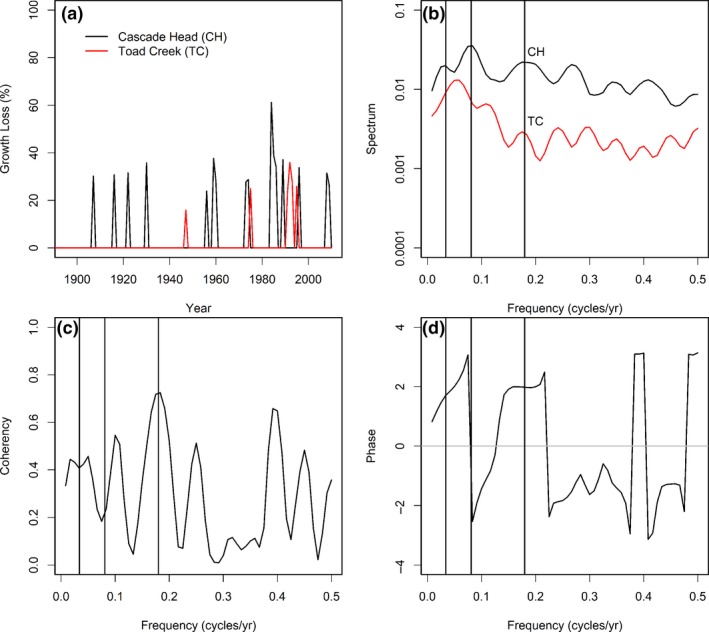
(a) Swiss needle cast indices of impact on earlywood growth for Cascade Head and Toad Creek are (c) coherent at higher frequencies but (d) out of phase. Toad Creek lags Cascade Head by several years at higher frequencies. (b) The power spectrum has a primary peak at a frequency of ~0.054 cycles/year for Toad Creek and ~0.083 cycles/year for Cascade Head

**Figure A4:**
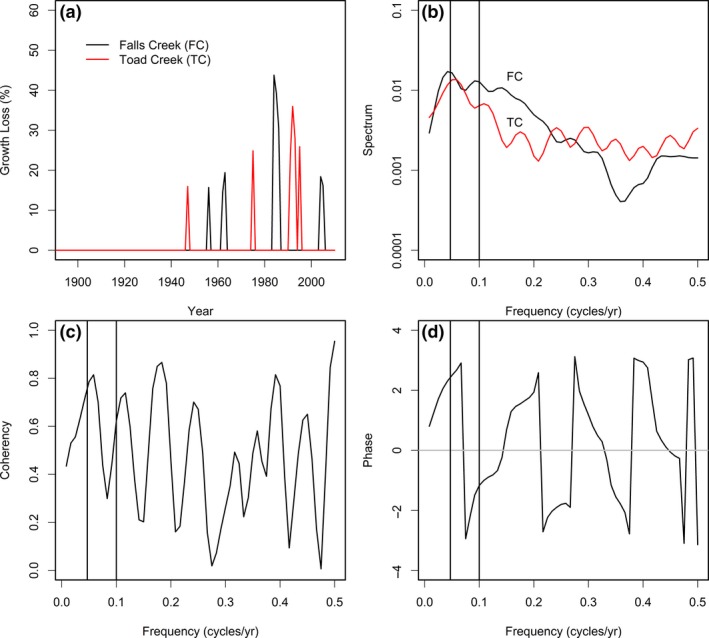
(a) Swiss needle cast indices of impact on earlywood growth for Falls Creek and Toad Creek are (c) coherent at lower frequencies but (d) out of phase. Toad Creek lags Falls Creek by several years at lower frequencies. (b) The power spectrum has a primary peak at a frequency of ~0.054 cycles/year for Toad Creek and ~0.042 cycles/year for Falls Creek

Cospectral analysis indicated that the SNC indices for CH, HCTL, and FC were coherent and in phase at the key frequencies (Figures A1 and A2). The SNC indices for several low‐ to mid‐elevations sites, CH and FC, and a higher‐elevation site, TC, were coherent at several key frequencies but were not in phase (Figures A3 and A4). In general, SNC outbreaks for SG and TC lagged the outbreaks for lower elevation sites by several years. The synchronization of SNC impact on Douglas‐fir across the landscape indicated that there were climate factors, which favored disease conditions for low‐ to midelevation sites in western Oregon (Figure 8). Peak SNC outbreaks were sometimes delayed by several years in the high Cascades where freezing winter temperatures slowed the development of pathogen population from reaching epidemic levels.

### 3.5 Seasonal climate factors

**Figure 9:**
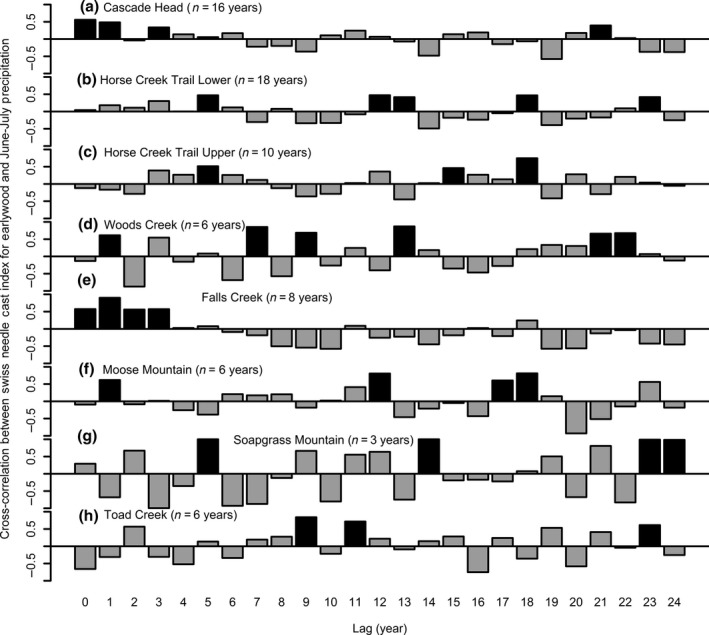
Cross‐correlation of Swiss needle cast (SNC) index of impact on earlywood growth with summer precipitation for eight study sites in western Oregon. The SNC index corresponds to the magnitude of the pulse intervention events identified from time‐series intervention analysis. The SNC index correlated best with precipitation in June and July for all sites. Correlations that are significantly different from zero at the 0.05 level of significance are denoted by a black bar

Cross‐correlation and canonical correlation analyses were used to determine the climatic factors associated with the SNC index for each site, focusing on EW growth for two coast (CH and HCTL) and two Cascade sites (FC and SG) where the magnitude and frequency of disease impacts and the statistical power of the test for correlation were greater. Disease impact related to winter temperature, summer precipitation, and temperature consistently across all study sites. The SNC index was generally positively correlated with current and past winter temperature and summer precipitation and negatively correlated with summer temperature. The impact of SNC on stem growth lagged the seasonal averages of winter and summer temperatures and summer precipitation by 0–24 years to varying degrees for all study sites. For three of the nine sites (CH, WC, and FC), the SNC index correlated best with summer precipitation lagged 1–3 years (Figure 9), similarly for summer and winter temperature (date not shown). In contrast, the SNC index for HCTL, HCTU, MM, TC, and SG correlated best with longer lags of summer precipitation, indicating a longer development period for a SNC outbreak event to occur.

Canonical correlation analysis was used to generate a climate index for each seasonal climate factor and study site based on a weighting scheme of current and previous years to optimize the correlation with the SNC index of impact (Table 4). Much stronger associations between the SNC index and seasonal temperature and precipitation were obtained when the climatic variables were averaged across current and past years than for individual years. Overall, canonical correlations with the EW SNC index were strong and ranged from 0.54 to 1.00 for winter temperature, 0.89 to 1.00 for summer precipitation, and −1.00 to −0.98 for summer temperature. Similarly for canonical correlations with LW SNC index. Summer precipitation and temperature were primary limiting factors of disease impacts for all sites, whereas winter temperature was a primary limiting factor for all sites except CH, HCTL, and possibly HCTU (Table 4).

**Table 4:** Canonical correlations (*r*_c_) between the Swiss needle cast index of impact and previous seasonal temperature and summer precipitation for nine study sites in western Oregon. Correlations in bold were statistically significant at the 0.05 level

Site	Growth period	No. of years	Winter temperature		June–July precipitation		June–July temperature	
			Lags	*r*_c_	Lags	*r*_c_	Lags	*r*_c_
Cascade Head	EW^a^	16	1–23	**0.74** c	0–21	**0.89**	3–24	**−0.98**
	LW^b^	8	4–14	**0.86** c	0–18	**1.00**	4–17	**−0.91**
Horse Creek Trail Lower	EW	18	1–11	**0.54** c	0–25	**0.91**	0–23	**−0.98**
	LW	9	1–15	0.46^c^	0–17	**1.00**	1–12	**−1.00**
Horse Creek Trail Upper	EW	10	0–11	**0.96** c	3–14	**1.00**	6–18	**−0.98**
	LW	8	5–20	**0.60** c	4–12	**0.97**	5–16	**−0.89**
Woods Creek	EW	6	0–11	**1.00** c	0–6	**1.00**	1–8	**−0.98**
	LW	5	0–11	**1.00** c	4–7	**1.00**	3–10	**−1.00**
Jackson Place	EW	1	NA	NA	NA	NA	NA	NA
	LW	5	6–10	**1.00** d	4–7	**1.00**	0–5	**−0.77**
Falls Creek	EW	8	0–12	**0.99** d	0–7	**1.00**	3–9	**−0.99**
	LW	6	1–11	**0.99** d	0–8	**1.00**	2–9	**−1.00**
Moose Mountain	EW	6	1–8	**1.00** d	1–11	**1.00**	0–10	**−1.00**
	LW	7	2–15	**1.00** d	2–14	**1.00**	3–7	**−0.58**
Soapgrass Mountain	EW	3	NA	NA	NA	NA	NA	NA
	LW	1	NA	NA	NA	NA	NA	NA
Toad Creek	EW	6	1–10	**1.00** e	2–11	**1.00**	0–12	**−1.00**
	LW	4	0–3	**1.00** e	0–2	**1.00**	0–4	**−1.00**

a Earlywood.

b Latewood.

c Winter temperature is calculated as the mean daily maximum temperature for December to February.

d Winter temperature is calculated as the mean daily maximum temperature for January to March.

e Winter temperature is calculated as the mean daily maximum temperature for February to April.

**Figure 10:**
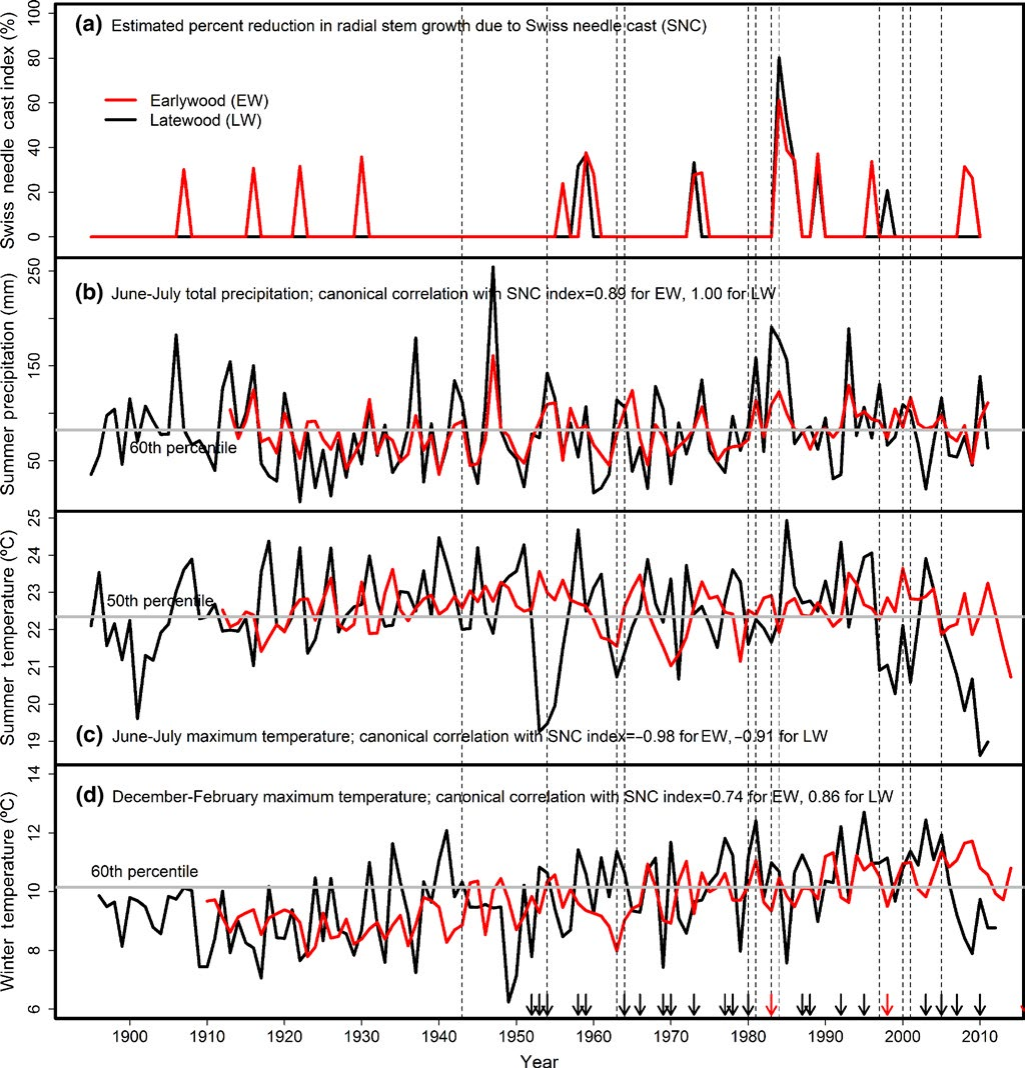
(a) Swiss needle cast (SNC) index of impact for Cascade Head is positively associated with (b) June–July precipitation and (d) December–February temperature and negatively associated with (c) June–July temperature. Fungal development was enhanced in years having warm winters and cool, wet summers. The years having all three seasonal climatic conditions favorable for Phaeocryptopus gaeumannii are indicated by vertical dashed lines. The SNC outbreak of 1984–1986 was preceded by several decades of warmer winters and cooler, wetter summers, most notably in 1983 during an extra strong El Niño event. The extra strong and weaker El Niño events since 1950 are indicated by red and black arrows, respectively. The canonical variables for winter and summer temperatures and summer precipitation (red line) are weighted averages of the current and previous 24 years

**Figure 11:**
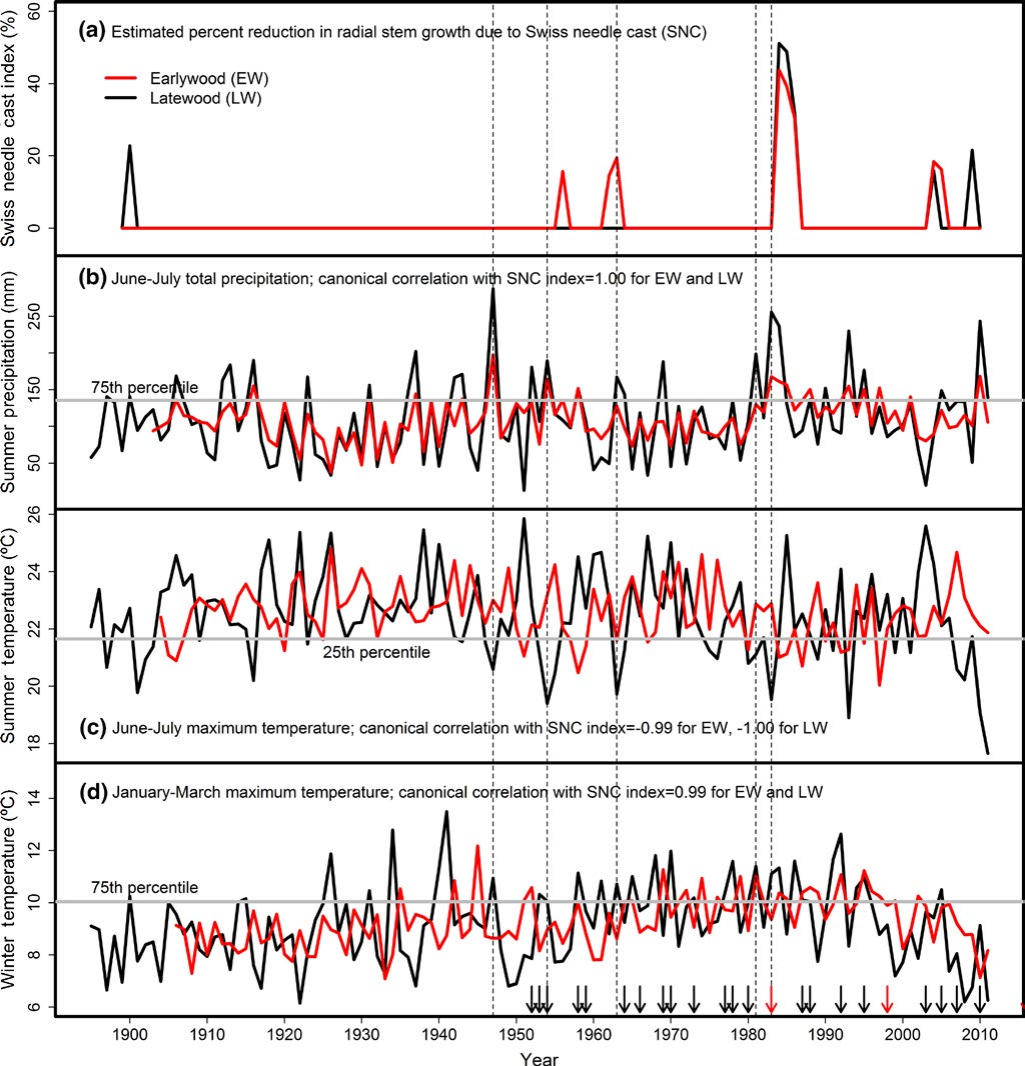
(a) Swiss needle cast (SNC) index of impact for Falls Creek is positively associated with (b) June–July precipitation and (d) January–March temperature and negatively associated with (c) June–July temperature. Fungal development was enhanced in years having warm winters and cool, wet summers. The years having all three seasonal climatic conditions favorable for Phaeocryptopus gaeumannii are indicated by vertical dashed lines. The SNC outbreak of 1984–1986 was preceded by several decades of warmer winters and cooler, wetter summers, most notably in 1983 during an extra strong El Niño event. The extra strong and weaker El Niño events since 1950 are indicated by red and black arrows, respectively. The canonical variables for winter and summer temperatures and summer precipitation (red line) are weighted averages of the current and previous 12 years

Variations in the intensity and duration of the episodic SNC outbreaks for individual sites were strongly associated with seasonal climatic factors lagged 0–24 years. The peak SNC outbreaks of 1959 and 1984–1986 were generally preceded by several decades of anomalously warm winters and cool, wet summers which allowed the pathogen abundance to build (Figures 10 and 11). The 1984–1986 outbreak was unprecedented and synchronous for low‐ to midelevation sites across the study area. The 1959 outbreak was less intense and shorter in duration than the 1984–1986 outbreak because the previous decades had fewer positive winter temperature and summer precipitation/temperature anomalies. For FC, there were 5 years having winter temperature >10.4°C (75th percentile), summer temperature <21.7°C (25th percentile), and summer precipitation >136 mm (75th percentile), of which 2 of 4 years since 1950 were classified as El Niño years (Figure 11). Since ~1960, there is an absence of synchronous outbreaks at mid‐ and high elevations in the Cascade Range (Figure 7). SNC outbreaks for SG and TC in the high Cascades have been intensifying since ~1960 in response to recent decades of warmer winters and episodic patterns of cooler, wetter summers (Figure 7h,i). For SG, there were 10 years with winter temperature >6.9°C (50th percentile), summer temperature <19.6°C (50th percentile), and summer precipitation >156 mm (75th percentile), of which 4 of 6 years since 1950 were classified as El Niño years.

## 4 DISCUSSION

Our dendrochronological reconstruction of SNC outbreaks in western Oregon indicates that outbreaks have been widespread and synchronous for the period 1895–2011. All sampled stands experienced significant radial growth reductions in Douglas‐fir that could not be accounted for by current and previous year seasonal climatic factors. The foliar disease, SNC, associated with the causal pathogen *P. gaeumannii*, is ubiquitous in western Oregon conifer forests and reduces the growth of Douglas‐fir by restricting gas and water exchange via stomatal occlusion and early needle abscission (Manter, Kelsey, & Stone, 2001). Unlike most forest pests and diseases specific to Douglas‐fir in the PNW, *P. gaeumannii* is found wherever its host is found (Boyce, 1940) and the severity of SNC symptoms varies annually in coastal Oregon where mild winters and cool, humid summers are most favorable for fungal development (Manter et al., 2001). In contrast, outbreaks of forest pests including the Douglas‐fir beetle (*Dendroctonus pseudotsugae* Hopkins), flatheaded fir borer (*Melanophila drummondi*), western spruce budworm (*Choristoneura occidentalis*), and Douglas‐fir tussock moth (*Orgyia pseudotsugata*) have historically been asynchronous and sporadic across western Oregon and Washington (Sheehan, Daterman, & Wenz 2004; Flower, Gavin, Heyerdahl, Parsons, & Cohn, 2014; Meigs, Kennedy, Gray, & Gregory, 2015; Raffa et al., 2008).

The forest health history of our permanent field sites is well documented based on long‐term dendrochronological records, field notes from monthly visits 1997 to present, complete tree mortality surveys, annual forest health surveys, and an ongoing epidemiological study of the canopy distribution of SNC. *P. gaeumannii* was present at low to damaging levels based on measurements of infection incidence and severity from Douglas‐fir needle samples collected at five of these sites in 2015 (Lan, Shaw, Beedlow, Lee, & Waschmann, 2017). We also found trees infected by laminated root rot (caused by the fungus *Phellinidium sulfurascens* (Pilat) YC Dai) and *Armillaria* root disease. Both laminated root rot (Thies & Sturrock, 1995) and *Armillaria* spp. (Shaw & Kile, 1991) are mortality agents of Douglas‐fir and reduce the growth of conifers. The mature Douglas‐fir trees for SG showed a slow decreasing century‐long growth trend similar to that of mountain pine (*Pinus mugo* Turra) trees in the Swiss National Park which were chronically infected with *Armillaria* (Cherubini et al., 2002). These low frequencies in endemic root diseases are unlikely to have caused the high‐frequency variability in stem growth of Douglas‐fir. In particular, the growth anomalies in 1984–1986 followed a wet period, whereas *Armillaria* spp. root disease is associated with hot, dry conditions (Shaw & Kile, 1991). Apart from SNC, there were no outbreaks of other forest pests and diseases specific to Douglas‐fir from 1997 to present, nor changes in tree mortality and regeneration rates and forest structure and composition associated with disturbance throughout the life history of conifer trees for these sites. Further, insect maps based on remote sensing indicate Douglas‐fir forests in western Oregon and Washington have not been affected by forest pests (Meigs et al., 2015; Raffa et al., 2008), which is consistent with our field observations.

Disease maps based on aerial surveys indicate a peak in SNC severity was observed in 2015 in Douglas‐fir forests west of the Coast Range crest of Oregon (Ritóková et al., 2016) and Washington (Ramsey et al., ). Several inland sites including a young Douglas‐fir plantation in Sweet Home, Oregon (N44°25′, W122°42′) (observation by members of the SNC Cooperative), and several of our permanent field sites (Lan et al., 2017) were also severely infected by SNC in 2015. However, SNC and other diseases have not been mapped in Douglas‐fir forests east of the Coast Range crest where disease symptoms of chlorosis and sparse canopies are subtle and cannot be diagnosed accurately by aerial surveys or remote sensing.

Because regional‐scale maps of forest diseases in the PNW are limited in time and space, we used tree‐ring records at the stand level to assess and identify the forest disturbances in Douglas‐fir forests in western Oregon based on their characteristics of frequency, duration, magnitude, and spatial extent. In coastal Oregon, evidence from dendrochronological studies shows that growth reductions associated with SNC are synchronous and episodic having primary periodicities of 20–40 years and secondary periodicities of 4–6 years in response to annual changes in climate and the abundance of the causal fungus (Lee et al., 2013, 2016). The episodic behavior of SNC impact on stem growth was most pronounced in infected Douglas‐fir trees in Tillamook, Oregon, where SNC reduced growth by 100% in 1984, 1996, and 2004 (Black et al., 2010). Within the SNC impact zone of Oregon, reductions in volume growth of young Douglas‐fir by SNC were estimated to range between 23% and 50% (Maguire, Mainwaring, & Kanaskie, 2011; Maguire et al., 2002).

Defoliation due to SNC is the cause of the synchronous cyclical patterns of aclimatic growth anomalies within the coastal fog zone (Black et al., 2010; Lee et al., 2013) and is the most likely cause of the concurrent patterns of cyclical growth anomalies across western Oregon. While there are several similarities between SNC and forest insects, SNC can be differentiated from other forest disturbance agents based on differences in their spatial extent, frequency, duration, and magnitude of impact, and potential to cause tree mortality. For example, several forest disturbance agents including Douglas‐fir tussock moth and western spruce budworm (WSB) differentially affect Douglas‐fir and western hemlock, have periodicities of ~30 years, and are widespread in the PNW, but differ from SNC in their potential to cause tree mortality and symptomatic response to climate. SNC and WSB (Alfaro, Berg, & Axelson, 2014; Axelson, Smith, Daniels, & Alfaro, 2015) differ in duration (1–3 years for SNC, 8–27 years for WSB), magnitude (WSB > SNC> 25% for climate), and spatial distribution (humid environments for SNC, drier environments for WSB). Furthermore, WSB, flatheaded fir borer, and bark beetles are commonly associated with drought stress (Flower et al., 2014; Furniss, 2014; Shaw, Oester, & Filip, 2009; Swetnam & Lynch, 1989, 1993), while SNC severity generally increases after several years of warm winters followed by wet summers (Lee et al., 2013). Some forest insects of Douglas‐fir (e.g., Douglas‐fir beetle) are ubiquitous in the PNW, but as a disturbance agent, their effects are sporadic and are associated with endogenous (e.g., population dynamics, host tree vigor, and susceptibility) and exogenous factors (e.g., climate) (Bentz et al., 2010; Raffa et al., 2008; Swetnam & Betancourt, 1998). Forest insects, alone or in combination with root diseases and drought stress, are very important causes of tree mortality and, consequently, are unlikely to have caused the cyclical patterns of aclimatic growth anomalies with no concurrent increase in the mortality rate.

Prolonged periods of high VPD conditions during the summer drought reduce stomatal conductance and can lead to carbon starvation and ultimately reduced annual stem growth, more so during a period of increased SNC severity when carbon uptake is further reduced by stomatal occlusion and early needle abscission. For five study sites (CH, FC, MM, SG, and TC), isotopic measurements of δ^13^C and δ^18^O discrimination in tree rings indicated that the growth anomalies in disturbed years could not be attributed to a physiological response to temperature and water stress but were consistent with a reduction in photosynthetic capacity by a loss of functioning stomata (data will be presented in a future manuscript).

Our tree‐ring chronologies of SNC outbreaks represent population cycles of the causal pathogen that are strongly associated with winter and summer temperatures and summer precipitation. Close correlations have been found between these seasonal climatic factors and epidemiological measurements of needle retention and *P. gaeumannii* abundance as well as growth impacts in the PNW (Hansen et al., 2000; Lee et al., 2013; Maguire et al., 2002; Manter et al., 2001; Winton, Manter, Stone, & Hansen, 2003; Winton, Stone, Watrud, & Hansen, 2002) and New Zealand (Watt, Stone, Hood, & Palmer, 2010). Low‐frequency variability in SNC impacts on Douglas‐fir growth is caused by the intensification of *P. gaeumannii* abundance to epidemic levels over several decades (Lee et al., 2013). The onset of a slow‐developing SNC outbreak occurs when the pathogen population reaches epidemic levels. High‐frequency variability in SNC impacts is the result of a delayed growth response to the infection of newly emerged needles each year and ensuing pathogen colonization in each needle age class.

### 4.1 Conceptual SNC disease cycle

**Figure 12:**
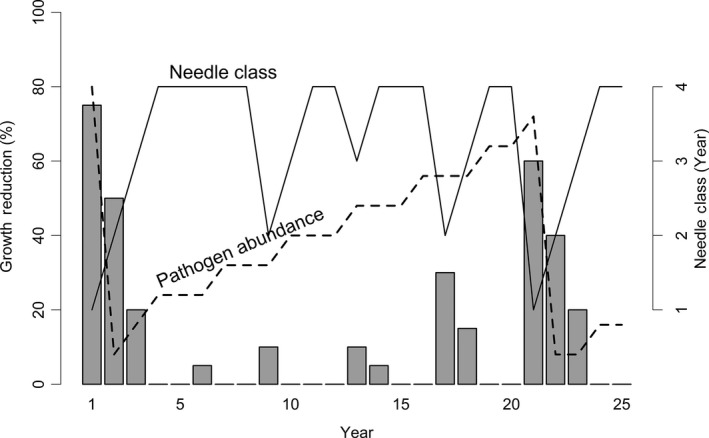
Conceptual model of Swiss needle cast impact on tree growth in association with the abundance of *Phaeocryptopus gaeumannii* and number of needle classes retained (Lee et al., 2013). The number of needle classes retained varies from one (when the tree is heavily infected) to four (least infected). Pathogen abundance increases from endemic (when 2‐year‐old and older needles are abscised) to epidemic levels (when tree is heavily infected) over several decades. The disease cycle begins anew with a peak reduction in growth when pathogen abundance reaches epidemic levels and is then reset to endemic levels following the early abscission of two‐year‐old and older needles. Growth reductions display 4‐ and ~20‐year periodicities because *P. gaeumannii* infects only the newly emerged needles at time of sporulation and has a 4‐year life cycle

We combined our dendroecological findings with the epidemiology of SNC to develop a conceptual model of the disease cycle driven by needle retention and fungal fruiting body abundance which have routinely been used as indices of disease severity (Hansen et al., 2000; Hood, 1982; Manter et al., 2005; Michaels & Chastagner, 1984). SNC reduces assimilation of carbon and tree diameter by stomatal occlusion and early needle abscission (Hansen et al., 2000; Manter, Bond, Kavanagh, Rosso, & Filip, 2000). Consequently, yearly changes in SNC impacts depend upon inoculum abundance, ascospore germination, and pathogen colonization in association with climatic conditions, which affect the proportion of stomata occluded and needle retention (Manter et al., 2005). Douglas‐fir trees on the coast typically retain up to 4 years of needles but may only have current and 1‐year‐old foliage due to premature needle abscission in severely affected plantations (Hansen et al., 2000; Maguire et al., 2002; Zhao et al., 2011). In our conceptual model, the disease cycle begins when pathogen abundance is at epidemic levels, resulting in loss of 2‐year‐old and older needles and a significant reduction in stem growth (Figure 12). The pathogen population will be reduced due to premature needle abscission resulting in fewer infected needles and a reduction in inoculum. Peak SNC outbreaks reduce tree growth for several consecutive years because photosynthetic capacity is restored to normal only after all needle classes have formed (Saffell et al., 2014). A delay of several years between inoculation and growth of the fungus and tree growth reduction is expected because the pathogen infects only the newly emerged needles (Hood & Kershaw, 1975; Stone, Capitano, et al., 2008). This lagged growth response to SNC is represented by a 4‐year periodicity in disease impacts (Figure 12). The slow buildup of pathogen abundance from endemic to epidemic levels over several generations is represented by a 20‐year periodicity. The conceptual disease model is illustrated as having a dominant periodicity of 20 years but, in reality, the primary periodicity varies by site and is as low as 6 years at Tillamook, Oregon, where more favorable climatic conditions allow the fungus to develop faster (Black et al., 2010; Lee et al., 2013; Stone, Coop, et al., 2008). Pseudothecia can be commonly found on 4‐ to 7‐year‐old needles in the Cascade Range of Oregon and Washington, and on 1‐ to 2‐year‐old needles in some areas of the Coast Range where pathogen dynamics are enhanced by more favorable climatic conditions (Stone, Coop, et al., 2008). Pathogen abundance is not reset to endemic levels by abscission of 2‐year‐old and older needles in areas where disease is constantly severe as indicated by a <10‐year disease cycle and the presence of pseudothecia on 1‐ to 2‐year‐old needles.

**Figure 13:**
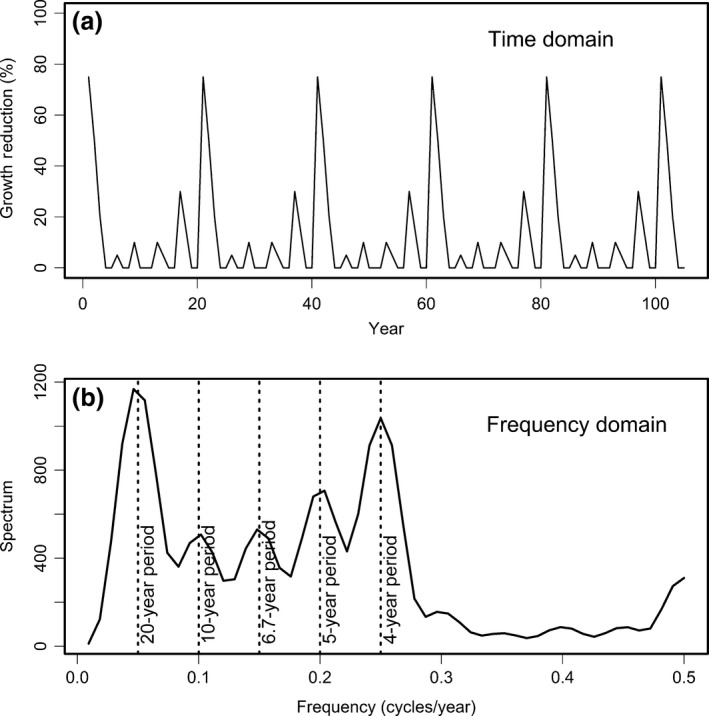
The sinusoidal pattern of Swiss needle cast (SNC) impact on growth of Douglas‐fir is represented by five repetitions of the 20‐year disease cycle. The SNC index has a primary periodicity of ~20 years and secondary periodicity of ~4 years as seen in the (a) time and (b) frequency domain. The total area under the spectrum is equal to the variance of the time series

We represent five replications of the conceptual disease cycle in the time and frequency domains (Figure 13). The dominant pattern in the disease cycle is a peak impact occurring every 20 years (Figure 13a) which is represented in the frequency domain by a peak in its spectrum at a frequency of 0.05 cycles/year (i.e., periodicity of 20 years) (Figure 13b). The spectrum also has a secondary periodicity of 4 years (frequency = 0.25 cycles/year) which is seen in the time domain as a periodic impact on growth every 4 years. The other local peaks in the spectrum occur at the harmonic periodicities, for example, twice and thrice the secondary periodicity. The spectrum in Figure 13b is the signature of SNC impact that is unique and can be used to identify a SNC disease cycle and separate the confounding effects of climate, SNC, and other forest disturbances at a site.

### 4.2 Spatiotemporal variability in SNC impacts

Detectable SNC impacts occur wherever Douglas‐fir is found in western Oregon and are greater and more frequent nearer the coast, at lower elevations on south‐facing aspects in the coastal fog zone (Manter, Winton, Filip, & Stone, 2003; Manter et al., 2005; Rosso & Hansen, 2003). Areas where environmental conditions are less favorable to the development of *P. gaeumannii* experience less frequent and less intense SNC outbreaks. The least severely impacted sites are found in warm, dry environments at low‐ to mid‐elevations in the Willamette Valley and Cascade Range and in cool environments in the high Cascades. Spatial variability in SNC severity depends upon site conditions, location, proximity to coast, and elevation (Rosso & Hansen, 2003; Stone, Coop, et al., 2008) and whether winter or summer conditions are more limiting (Zhao et al., 2011).

SNC reduces Douglas‐fir growth relative to other tree species and therefore can affect competitive outcomes between tree species. This is very well illustrated by the Sitka spruce (*Picea sitchensis*) vegetation zone of the Coast Range of Oregon and Washington where *P. gaeumannii* is most abundant and Douglas‐fir is a minor species compared to western hemlock, even though Douglas‐fir grow much more rapidly than hemlock where environmental conditions for *P. gaeumannii* are less favorable. The composition of the Sitka spruce vegetation zone is distinct and shaped not only by climate favorable to growth of Sitka spruce and western hemlock but also to *P. gaeumannii* and its negative effect on the competitive ability of Douglas‐fir.

### 4.3 SNC outbreaks are influenced by broad‐scale forcing factors

The intensity and duration of SNC outbreaks are influenced by multidecadal seasonal temperature and precipitation trends which are modulated by several broad‐scale forcing factors including the El Niño‐Southern Oscillation (ENSO), the Pacific‐North American (PNA) pattern, and the Pacific Decadal Oscillation (PDO) (Lee et al., 2013). Long‐term PNW temperature records show an accelerated warming trend since 1970, most notably in the winter months (December–February) resulting in a longer freeze‐free season and warmer minimum temperatures in winter (Abatzoglou, Rupp, & Mote, 2014). PNW precipitation records show a downward trend during a strong warm phase of the PDO (1925–1946) followed by a heterogeneous but positive trend in spring precipitation during and after a strong cool phase of the PDO (1947–1978) (Abatzoglou & Redmond, 2007). These multidecadal trends in seasonal temperature and precipitation are reflected in the lower intensity of SNC outbreaks prior to 1950 relative to outbreaks after 1950.

Swiss needle cast outbreaks tend to be more severe during periods of relatively warm winter and cool, wet summer conditions (Lee et al., 2013). Synchrony in SNC impacts is attributed to a single or several consecutive extreme climate events toward the end of a disease cycle when the amount of inoculum is high. The wettest 25‐year period was 1960–1984 with most years above the historical water year average precipitation (Abatzoglou et al., 2014). The warm winters and cool, wet summers in this period allowed *P. gaeumannii* to develop rapidly, resulting in a widespread SNC outbreak in 1984–1986. Following the SNC outbreak initiated in 1983, there was a delay of 1 year before the reduction in photosynthate production due to stomatal occlusion and early needle abscission was fully expressed in stem growth. The anomalously cool, wet summer in 1983 occurred during an extra strong El Niño event. However, broad‐scale forcing factors such as ENSO and PNA modulate seasonal temperature patterns but not precipitation patterns (Abatzoglou et al., 2014). Long‐term climate records show an increase in spring precipitation and a decrease in summer and autumn precipitation, indicating that summer conditions may become more of a limiting factor for *P. gaeumannii* in warm, dry environments with a changing climate.

Rapid warming in the PNW during December–February and March–May since ~1950 has been attributed to ENSO and PNA (Abatzoglou & Redmond, 2007; Abatzoglou et al., 2014). The warmest 10‐year period was 1998–2007 with most years above the historical annual mean temperature. With warmer winters, SNC impacts are increasing in mature closed‐canopy Douglas‐fir stands on the east slopes of the Coast Range and in the high Cascades as SNC impacts on Douglas‐fir growth were greatest in the 1990s for these sites. Warming winters are expected to intensify SNC outbreaks more at high elevations and higher latitudes because the proliferation of pseudothecia is highly sensitive to small increases in winter temperature above the temperature threshold of ~4°C for fungal growth (Stone, Coop, et al., 2008). A temperature threshold of 4°C is consistent with the spatial shift in the association between SNC impact and winter temperature in December–February for the Coast Range to February–March in the high Cascades. Long‐term observations show an increase in the length of the freeze‐free season since 1970 by an average rate of 0.5 weeks per decade (Abatzoglou et al., 2014). This increase in winter temperature and freeze‐free days is expected to continue in the 21st century, resulting in a proliferation of pseudothecia by extending the growing season and shifting temperatures above the threshold.

Swiss needle cast has been and will continue to be an important forest disease of Douglas‐fir in the PNW, New Zealand, Europe, Chile, Australia, and elsewhere in light of the broader spatial extent of SNC impact beyond the coastal fog zone. Douglas‐fir forests will respond differently to the interacting effects of SNC and climate, depending upon location and site condition, as temperatures rise and precipitation patterns shift toward wetter winters and springs and drier summers. Higher winter temperatures are expected to enhance pseudothecia production, more so in cool environments, but warmer, drier summers are expected to inhibit *P. gaeumannii* development and growth by reducing the ascospore germination rate at time of sporulation. Summer precipitation is strongly associated with the magnitude and frequency of SNC impacts on Douglas‐fir growth for all sites. Under future climate change scenarios, SNC impacts are likely to increase in regions where summer precipitation frequently exceeds the *P. gauemannii*‐limiting threshold of 110 mm (Hood, 1982) and decrease elsewhere where the summer precipitation threshold is seldom exceeded. Because the greatest warming due to climate change is predicted to occur in the winter and summer (Mote, Abatzoglou, & Kunkel, 2013), SNC in the PNW is expected to intensify in frequency and magnitude at higher elevations and/or higher latitudes along the coast and inland where current winter temperatures are a primary limiting factor to fungal growth.

## CONFLICT OF INTEREST

None declared.

## AUTHOR CONTRIBUTIONS

EHL, PAB, and DTT conceived of and designed the study. RSW provided technical support for the network of long‐term monitoring field sites. EHL, PAB, RSW, SC, MB, and CC collected the field data and CC processed the tree core samples in the laboratory. EHL and CW analyzed the data. EHL wrote the first draft of the manuscript, and all authors contributed substantially to revisions.

## Figures and Tables

**Figure A5:**
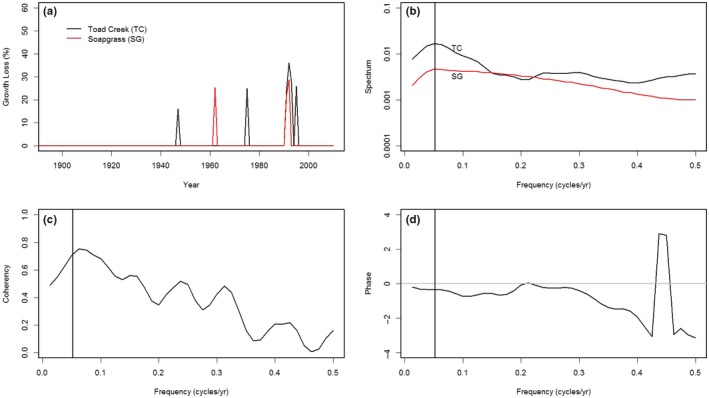
(a) Swiss needle cast (SNC) indices of impact on earlywood growth for Toad Creek and Soapgrass Mountain are (c) coherent at higher and lower frequencies and (d) in phase. The power spectrum has a primary peak at a frequency of ~0.054 cycles/year for Toad Creek, whereas the power spectrum for Soapgrass Mountain has a broader peak at frequencies ranging from 0.05 to 0.18 cycles/year

## Appendix 1

### Time Series Models with Pulse Interventions

The site‐specific growth‐climate‐disturbance models reported by Lee et al. (2016) were modified to simplify the earlywood and latewood growth response to temperature and water stress and to better separate the confounding effects of climate and forest disturbances on radial stem growth. Supplementary dual isotope discrimination data and nonhost chronologies that were not previously available were used in this study to detect growth anomalies that represented divergences between the host chronology and the climate proxies including the nonhost chronology and isotopic measurements of δ^13^C and δ^18^O discrimination in tree rings. Years were classified into two categories (no suppression and suppression) based on outlier detection tests using time‐series intervention analysis. This resulted in more parsimonious models than the previous time series models that classified years into three categories (no suppression, suppression, release) (Lee et al., 2016). Furthermore, we used a more stringent test for outlier detection and supplemental data to reconstruct the history of forest disturbance impacts on Douglas‐fir growth at the stand level. Several growth anomalies were classified as disturbance years based on observed divergences between the host and climate proxies when the Wald test for outliers was not statistically significant based on the Bonferroni adjustment and an experiment‐wise error rate of 0.05. The growth‐climate‐disturbance models by site and growth period are presented in Table A1.

### Cospectral Analysis of SNC Impact

Swiss needle cast indices of impact for CH and FC display pseudoperiodic patterns having a primary periodicity of ~30 years and a secondary periodicity of ~6.7 years (Figure A1b). The SNC chronologies for CH and FC are coherent and in phase at the key frequencies (Figure A1c&d). To a lesser extent, SNC impacts for HCTL and FC are coherent and in phase (Figure A2). SNC impacts for CH and TC are coherent at several key frequencies but out of phase (Figure A3). SNC impacts for TC lag those for CH by several years. Similarly, SNC impacts for FC and TC are coherent at the key frequencies but SNC impacts for TC lag those for FC by several years (Figure A4). SNC impacts for SG and TC are coherent and in phase at the primary frequency of ~0.054 cycles/year (Figure A5).

**Table A1:** Growth response of Douglas‐fir to seasonal climatic variables based on time‐series intervention model of earlywood and latewood ring width series at (A) Cascade Head, (B) Horse Creek Trail Lower, (C) Horse Creek Trail Upper, (D) Woods Creek, (E) Jackson Place, (F) Falls Creek, (G) Moose Mountain, (H) Soapgrass Mountain, and (I) Toad Creek. Maximum likelihood estimates and the associated standard errors of the slopes for the measured climate variables are reported. Coefficients in bold are significantly different from 0 at the 0.05 level of significance

Variable or parameter	Earlywood			Latewood		
	Season	Estimate	Standard error	Season	Estimate	Standard error
(A) Cascade Head, 1895–2010, *R* ^2^ = 0.76						
Intercept		−1.17	1.18		−**4.24**	1.56
Prior‐year DPD	June–October	−0.0091	0.0083		−0.0030	0.0096
Winter Temp				November–January	**0.0187**	0.0044
Summer Temp Linear	June–July	0.1149	0.1074	July–September	**0.3462**	0.1319
Summer Temp Quadratic	June–July	−0.0026	0.0024	July–September	−**0.0073**	0.0028
Pulse 1907		−**0.1560**	0.0479			
Pulse 1916		−**0.1597**	0.0491			
Pulse 1922		−**0.1648**	0.0499			
Pulse 1930		−**0.1923**	0.0483			
Pulse 1956		−**0.1189**	0.0488			
Pulse 1958					−**0.1657**	0.0511
Pulse 1959		−**0.2053**	0.0508		−**0.1978**	0.0489
Pulse 1960		−**0.1444**	0.0520			
Pulse 1973		−**0.1415**	0.0541		−**0.1754**	0.0479
Pulse 1974		−**0.1472**	0.0511			
Pulse 1984		−**0.4115**	0.0564		−**0.7027**	0.0485
Pulse 1985		−**0.2135**	0.0557		−**0.3228**	0.0507
Pulse1986		−**0.1817**	0.0566		−**0.1662**	0.0500
Pulse 1989		−**0.2014**	0.0567		−**0.1563**	0.0476
Pulse 1991		−**0.0941** a	0.0560			
Pulse 1993					−**0.1187** a	0.0477
Pulse 1996		−**0.1786**	0.0490			
Pulse 1998					−**0.1011**	0.0496
Pulse 2008		−**0.1644**	0.0591			
Pulse 2009		−**0.1335**	0.0610			
Seasonal AR, θ_1_		0.26			0.20	
Seasonal AR, θ_2_		−0.55				
Seasonal AR, θ_3_		0.07				
Seasonal AR, θ_4_		−0.32				
Regular AR, ρ_1_		0.50			0.49	
σ		0.0606			0.0457	
(B) Horse Creek Trail Lower, 1895–2011, *R* ^2^ = 0.72						
Intercept		−**3.83**	0.94		−0.24	0.16
Prior–year DPD	June–October	−**0.0266**	0.0089	June–October	−0.0121	0.0143
Summer DPD				July–September	−0.0268	0.0141
Summer Temp Linear	June–July	**0.3651**	0.0859	July–September	**0.0231**	0.0087
Summer Temp Quadratic	June–July	−**0.0082**	0.0019			
Summer PDSI	July	**0.0134**	0.0025	August	**0.0127**	0.0034
Fog Frequency	June–July	−**0.0451**	0.0157	July–September	−**0.0650**	0.0180
Pulse 1902		−**0.1665**	0.0404			
Pulse 1907		−**0.1110**	0.0416			
Pulse 1918					−**0.2359** a	0.0612
Pulse 1919		−**0.1431**	0.0407			
Pulse 1926		−**0.0911** a	0.0410		−**0.2813** a	0.0591
Pulse 1930		−**0.1087**	0.0403			
Pulse 1932					−**0.1862**	0.0605
Pulse 1933		−**0.1344**	0.0406			
Pulse 1937		−**0.1216** a	0.0416		−**0.1959** a	0.0599
Pulse 1953		−**0.0984**	0.0454			
Pulse 1956		−**0.1029**	0.0417			
Pulse 1957		−**0.0978**	0.0431			
Pulse1958					−**0.1414**	0.0604
Pulse 1959		−**0.2219**	0.0426		−**0.2966**	0.0603
Pulse 1960		−**0.1985**	0.0431			
Pulse 1962		−**0.1091**	0.0431			
Pulse 1984		−**0.2144**	0.0438		−**0.1827**	0.0603
Pulse 1985		−**0.0849**	0.0444		−**0.3125**	0.0601
Pulse 1986		−**0.2400**	0.0428		−**0.1562**	0.0614
Pulse 1991		−**0.1457**	0.0406		−**0.1205**	0.0618
Pulse 1995					−**0.1742**	0.0600
Pulse 1996		−**0.1342**	0.0424			
Pulse 2000		−**0.1063**	0.0399			
Pulse 2005		−**0.1233**	0.0438		−**0.1144**	0.0631
Seasonal AR, θ_1_		−0.28				
Seasonal AR, θ_2_		0.36				
Regular AR, ρ_1_		0.36			0.66	
σ		0.0452			0.0532	
(C) Horse Creek Trail Upper, 1895–2011, *R* ^2^ = 0.60						
Intercept		−**4.20**	1.27		−0.47	1.34
Prior‐year DPD	June–October	−0.0131	0.0102	June–October	−0.0067	0.0073
Winter Temp	January	0.0051	0.0031	February–April	**0.0145**	0.0030
Summer Temp Linear	June–July	**0.3839**	0.1151	July–September	0.0349	0.1130
Summer Temp Quadratic	June–July	−**0.0087**	0.0026	July–September	−0.0008	0.0023
Summer PDSI	July	0.0050	0.0031	August	**0.0041**	0.0022
Fog Frequency	June–July	0.0336	0.0222			
Pulse 1918					−**0.1614** a	0.0433
Pulse 1933					−**0.1148**	0.0431
Pulse 1951		−**0.1598**	0.0558		−**0.1719**	0.0422
Pulse 1952		−**0.1450**	0.0557			
Pulse 1956		−**0.1219**	0.0541			
Pulse 1959		−**0.1530**	0.0546		−**0.2022**	0.0423
Pulse 1968		−**0.1440**	0.0550			
Pulse 1976		−**0.1258**	0.0544			
Pulse 1984		−**0.1383**	0.0560		−**0.1007**	0.0433
Pulse 1985		−**0.1338**	0.0605		−**0.1549**	0.0438
Pulse 1986		−**0.1645**	0.0558		−**0.1464**	0.0432
Pulse 1991		−**0.1663** a	0.0544		−**0.1557** a	0.0447
Pulse 1999					−**0.1102**	0.0442
Pulse 2000		−**0.1585**	0.0539			
Pulse 2011					−**0.1339**	0.0473
Seasonal AR, θ_1_		0.19			−0.14	
Regular AR, ρ_1_		0.40			0.55	
σ		0.0503			0.0392	
(D) Woods Creek, 1895–2012, *R* ^2^ = 0.67						
Intercept		**0.11**	0.04		0.09	0.04
Trend 1980–2012		−**0.0013**	0.0006		−**0.0010**	0.0003
Prior‐year DPD	June–September	−**0.0226**	0.0052	June–October	−**0.0149**	0.0046
Winter Temp	November–February	**0.0107**	0.0034	November–February	**0.0111**	0.0035
Summer DPD	May–June	−**0.0178**	0.0044	June–August	−**0.0193**	0.0051
Summer PDSI	June–July	**0.0085**	0.0022	July–August	**0.0066**	0.0021
Pulse 1906					−**0.0639** a	0.0336
Pulse 1953		−**0.1010**	0.0357		−**0.0911**	0.0334
Pulse 1959		−**0.1071**	0.0350		−**0.2109**	0.0329
Pulse 1965		−**0.1616** a	0.0356		−**0.0965**	0.0328
Pulse 1985					−**0.0639**	0.0338
Pulse 1989					−**0.0656** a	0.0351
Pulse 1991		−**0.2072** a	0.0381		−**0.1826** a	0.0363
Pulse 1993		−**0.0897**	0.0389			
Pulse 1995		−**0.0834**	0.0366		−**0.0812**	0.0337
Pulse 1996		−**0.1737**	0.0357			
Pulse 2009					−**0.0776**	0.0352
Pulse 2010		−**0.0974**	0.0358			
Seasonal AR, θ_1_		0.10			0.18	
Seasonal AR, θ_2_		0.29			−0.37	
Regular AR, ρ_1_		0.28			0.46	
σ		0.0358			0.0353	
(E) Jackson Place, 1895–2012, *R* ^2^ = 0.79						
Intercept		**0.57**	0.11		0.16	0.08
Trend 1900−2012		−**0.0035**	0.0003		−**0.0029**	0.0003
Prior‐year DPD	June–September	−**0.0300**	0.0102			
Winter Temp	November	**0.0161**	0.0046	February–March	**0.0219**	0.0043
Summer DPD	May–July	−**0.0210**	0.0092	June–August	−**0.0265**	0.0095
Summer PDSI				July–September	**0.0117**	0.0044
Summer Precip				July–August	**0.0011**	0.0003
Pulse 1901					−**0.1345**	0.0584
Pulse 1904					−**0.1220**	0.0605
Pulse 1908					−**0.1408**	0.0600
Pulse 1918		−**0.2272** a	0.0701		−**0.2118** a	0.0588
Pulse 1919		−**0.1447** a	0.0678			
Pulse 1937		−**0.1556** a	0.0648			
Pulse 1939					−**0.1127** a	0.0599
Pulse 1956					−**0.1494**	0.0589
Pulse 1959					−**0.1267** a	0.0589
Pulse 1968					−**0.1933**	0.0715
Pulse 1993		−**0.1586**	0.0650			
Seasonal AR, θ_1_		0.37			0.44	
Regular AR, ρ_1_		0.30			0.23	
σ		0.0690			0.0656	
(F) Falls Creek, 1899–2010, *R* ^2^ = 0.75						
Intercept		**0.086**	0.049		**0.298**	0.077
Trend 1990–2011		−**0.0062**	0.0008		−**0.0077**	0.0010
Prior‐year DPD	July–September	−0.0073	0.0042	October–November	−**0.0149**	0.0045
Winter Temp	November–February	**0.0089**	0.0028	January–March	**0.0117**	0.0028
Summer DPD	May–June	−**0.013**	0.004			
Summer Temp				July–August	−**0.0135**	0.0029
Summer Precip				June–July	0.0015	0.0008
Pulse 1900					−**0.1123**	0.0405
Pulse 1917					−**0.0826** a	0.0414
Pulse 1918		−**0.1280** a	0.0336			
Pulse 1931					**0.2024**	0.0409
Pulse 1956		−**0.0742**	0.0329			
Pulse 1962		−**0.0683**	0.0333			
Pulse 1963		−**0.0937**	0.0340			
Pulse 1984		−**0.2500**	0.0342		−**0.3112**	0.0417
Pulse 1985		−**0.2165**	0.0340		−**0.2912**	0.0415
Pulse 1986		−**0.1581**	0.0336		−**0.1676**	0.0406
Pulse 2004		−**0.0884**	0.0343		−0.0751	0.0407
Pulse 2005		−**0.0768**	0.0353			
Pulse 2009					−**0.1056**	0.0427
Seasonal AR, θ_1_		0.16			0.15	
Regular AR, ρ_1_					0.38	
σ		0.0291			0.0375	
(G) Moose Mountain, 1903–2010, *R* ^2^ = 0.72						
Intercept		**0.22**	0.06		**0.25**	0.08
Trend 1990–2010		−**0.0062**	0.0010		−**0.0082**	0.0010
Prior‐year DPD	June–October	−**0.0244**	0.0055	June–October	−**0.0163**	0.0067
Winter Temp	November–February	**0.0144**	0.0033	November–February	**0.0171**	0.0040
Summer DPD	May–July	−**0.0124**	0.0050	July–August	−**0.0232**	0.0064
Summer PDSI				July–August	**0.0068**	0.0029
Summer Precip				July–August	**0.0043**	0.0020
Pulse 1903					−**0.0868**	0.0467
Pulse 1904		−**0.0802**	0.0389		−**0.2070**	0.0465
Pulse 1917					−**0.0998** a	0.0469
Pulse 1918		−**0.0871** a	0.0380		−**0.0953** a	0.0475
Pulse 1924		−**0.1300**	0.0386			
Pulse 1945					−**0.1476**	0.0466
Pulse 1951		−**0.1028**	0.0376			
Pulse 1953		−**0.1241**	0.0388		−**0.1359**	0.0472
Pulse 1954		−**0.1250**	0.0386			
Pulse 1959					−**0.0892** a	0.0467
Pulse 1963		−**0.0910**	0.0381			
Pulse 1965					−**0.0853**	0.0479
Pulse 1968					−**0.1479**	0.0499
Pulse 1970					−**0.1613**	0.0475
Seasonal AR, θ_1_		0.29				
Regular AR, ρ_1_		0.23			0.55	
σ		0.0375			0.0419	
(H) Soapgrass Mountain, 1895–2010, *R* ^2^ = 0.85						
Intercept		**0.59**	0.06		**0.53**	0.07
Trend 1904–2010		−**0.0033**	0.0001		−**0.0021**	0.0002
Prior‐year DPD	June–October	−**0.0291**	0.0062	June–October	−**0.0284**	0.0063
Winter Temp	November–February	**0.0179**	0.0041	November–February	**0.0174**	0.0042
Summer DPD	June–July	−**0.0082**	0.0048	July–September	−**0.0172**	0.0054
Summer Precip				June–July	**0.0017**	0.0008
Pulse 1937		−**0.1902** a	0.0486			
Pulse 1951		−**0.1652** a	0.0494			
Pulse 1952		−**0.1554** a	0.0490			
Pulse 1953		−**0.1498** a	0.0493		−**0.1185** a	0.0505
Pulse 1954		−**0.1685** a	0.0488		−**0.1692** a	0.0510
Pulse 1962		−**0.1262**	0.0495			
Pulse 1989		−**0.1303** a	0.0499			
Pulse 1991		−**0.1077**	0.0485		−**0.1036**	0.0509
Pulse 1992		−**0.1464**	0.0493			
Regular AR, ρ_1_		0.38			0.69	
σ		0.0446			0.0477	
(I) Toad Creek, 1895–2010, *R* ^2^ = 0.62						
Intercept		**0.21**	0.05		**0.15**	0.05
Trend 1987–2010		−0.0088	0.0049		−**0.0484**	0.0126
Prior‐year DPD	June–October	−**0.0162**	0.0053	June–October	−**0.0168**	0.0052
Winter Temp	November–February	**0.0180**	0.0032	November–February	**0.0119**	0.0034
Summer DPD	May–July	−**0.0242**	0.0051	July–September	−0.0085	0.0045
Pulse 1917					−**0.0928** a	0.0404
Pulse 1918		−**0.1419** a	0.0392		−**0.1444** a	0.0407
Pulse 1947		−**0.0755**	0.0387			
Pulse 1953		−**0.1158** a	0.0390			
Pulse 1970					−**0.1023**	0.0419
Pulse 1975		−**0.1242**	0.0407			
Pulse 1977					−**0.0862**	0.0417
Pulse 1987					−**0.0869** a	0.0410
Pulse 1988					−**0.0844** a	0.0423
Pulse 1989		−**0.0986** a	0.0394		−**0.0783** a	0.0417
Pulse 1991		−**0.1184**	0.0403		−**0.1137**	0.0414
Pulse 1992		−**0.1937**	0.0438		−**0.1152**	0.0418
Pulse 1993		−**0.1423**	0.0427			
Pulse 1995		−**0.1302**	0.0404			
Seasonal AR, θ_1_		0.29				
Regular AR, ρ_1_		0.31			0.36	
σ		0.0388			0.0365	

a Low growth anomaly for Douglas‐fir was concurrent with low growth anomaly for western hemlock and was not attributed to Swiss needle cast. The low growth anomaly in 1918 was likely caused by an exceptionally hot dry summer as indicated by a record high dewpoint deficit in June and PDSI < −2.4 (i.e., moderate‐to‐severe drought) for June–September.
